# The Ectopic Overexpression of the Cotton *Ve1* and *Ve2*-Homolog Sequences Leads to Resistance Response to Verticillium Wilt in *Arabidopsis*

**DOI:** 10.3389/fpls.2017.00844

**Published:** 2017-05-29

**Authors:** Jieyin Chen, Nanyang Li, Xuefeng Ma, Vijai K. Gupta, Dandan Zhang, Tinggang Li, Xiaofeng Dai

**Affiliations:** ^1^Laboratory of Cotton Diseases, The Institute of Food Science and Technology, Chinese Academy of Agricultural SciencesBeijing, China; ^2^Department of Chemistry and Biotechnology, ERA Chair of Green Chemistry, School of Science, Tallinn University of TechnologyTallinn, Estonia

**Keywords:** cotton, *Verticillium* wilt, receptor-like proteins, virus-induced gene silencing, transgenic, microarray analysis

## Abstract

*Verticillium* wilt, caused by the *Verticillium dahliae* phytopathogen, is a devastating disease affecting many economically important crops. A receptor-like protein (RLP) gene, *Ve1*, has been reported to confer resistance to *V. dahliae* in tomato plants, but few genes have been found to be involved in cotton *Verticillium* wilt resistance. Here, we cloned two *RLP* gene homologs, *Gossypium barbadense* resistance gene to *Verticillium dahliae* 1 (*GbaVd1*) and *GbaVd2*, from the *Verticillium* wilt-resistant cultivar *G. barbadense* cv. Hai7124. *GbaVd1* and *GbaVd2* display sequence divergence, but both encode typical RLPs. Virus-induced gene silencing of *GbaVd1* or *GbaVd2* compromised the resistance of cotton to *V. dahliae*, and both genes conferred *Verticillium* wilt resistance after interfamily transfer into *Arabidopsis*. Microarray analysis revealed that *GbaVd1* and *GbaVd2* participate in *Verticillium* wilt resistance in *Arabidopsis* through activation of defense responses, including the endocytosis process, signaling factors, transcription factors and reinforcement of the cell wall, as demonstrated by lignification in *Arabidopsis* transgenic plants. In addition, microarray analysis showed that *GbaVd1* and *GbaVd2* differentially mediate resistance signaling and activation of defense responses after overexpression in *Arabidopsis*. Thus, *GbaVd1* and *GbaVd2* encode RLPs and act as disease resistance genes that mediate the defense response against *V. dahliae* in cotton.

## Introduction

*Verticillium dahliae* Kleb. is a soil-borne vascular wilt fungus characterized by a broad host range of over 200 dicotyledonous species (Fradin and Thomma, 2006; Klosterman et al., 2009). Improving genetic resistance is a preferred method to manage *Verticillium* wilt in several crop plants (Schaible et al., 1951; Putt, 1964; Huang, 2003; Simko et al., 2004; Bolek et al., 2005). However, only the tomato *Verticillium* (*Ve*) locus has been efficiently used in molecular breeding (Kawchuk et al., 2001; Fradin et al., 2009). The tomato *Ve* locus contains two closely linked, inversely oriented genes: *Ve1* and *Ve2*. These genes encode cell surface receptors that belong to a group of extracellular receptor-like proteins (RLPs), which typically include leucine-rich repeat (LRR) domains, a single-pass transmembrane domain, and a short cytoplasmic tail that lacks obvious motifs for directly activating intracellular signal transduction (Kawchuk et al., 2001). The *RLP* genes involved in plant disease resistance have been widely identified and include tomato leaf mold resistance *Cf* genes (Dixon et al., 1996, 1998; Parniske et al., 1997; Rivas and Thomas, 2005), the apple scab resistance gene *HcrVf2* (Vinatzer et al., 2001), and the tomato *LeEix1* and *LeEix2* genes encoding the fungal elicitor ethylene-inducing xylanase of *Trichoderma viride* (Ron and Avni, 2004).

The *Ve* gene mediates disease resistance against *V. dahliae* race 1 strains, but is not functional against race 2 stains (Schaible et al., 1951; Fradin et al., 2009). More recent research has found that only *Ve1*, but not *Ve2*, has functional *Verticillium* wilt resistance activity in tomato plants (Fradin and Thomma, 2006; Fradin et al., 2009). Additionally, the interfamily transfer of *Ve1* to *Arabidopsis thaliana* provides fully functional *Verticillium* wilt resistance (Fradin et al., 2011). Comparative genomic analysis of race 1 and race 2 strains has identified an effector named Ave1, a small secreted protein with four cysteines that contributes to full virulence in tomato plants lacking *Ve1* but acts as an avirulence factor recognized by *Ve1*, and activates *Ve1*-mediated resistance (de Jonge et al., 2012).

The resistance mechanism of RLPs has been well described in the *Cf* genes, which mediate tomato resistance to *Cladosporium fulvum*. RLPs use LRR domains to recognize effectors that are secreted by pathogens and activate the defense response through the cooperation of the cytoplasmic tail with diverse signal factors (Joosten and de Wit, 1999). Several studies have shown that a signal factor binding to the cytoplasmic tail is required for RLP function, such as the interactions of vesicle-associated protein 27 (VAP27) and *Cf-9* interacting thioredoxin (CITRX) with the *Cf-9* cytoplasmic tail (Laurent et al., 2000; Rivas et al., 2004). Moreover, many signal factors have also been shown to participate in defense responses mediated by RLPs, such as the protein kinase Avr9/Cf-9 induced kinase 1 (ACIK1) (Rowland et al., 2005), the NB-LRR required for HR-associated cell death-1 (NRC1) (Gabriëls et al., 2006, 2007), the U-box protein of Cys, Met, Pro, and Gly protein 1 (CMPG1) (González-Lamothe et al., 2006), the *Lycopersicon esculentum* mitogen-activated protein kinase 1 (LeMPK1), LeMPK2, and LeMPK3 (Stulemeijer et al., 2007), and the F-box protein Avr9/Cf-9 rapidly elicited protein 189 (ACRE189) (van den Burg et al., 2008). The endocytosis signal motif in the cytoplasmic tail also plays an important role in the signal transduction mediated by RLPs. Vesicle-associated protein 27 (VAP27) possesses an endocytosis signal with a KKX motif that is involved in membrane trafficking (Theriot, 1996; Laurent et al., 2000; Bonifacino and Traub, 2003). LeEix2 contains a YXXø (ø is an amino acid with a hydrophobic side chain) motif that stimulates RLP-mediated endocytosis, and its ability to transport the elicitor into the cytoplasm via endocytosis is lost after deletion of this motif (Ron and Avni, 2004). Genetic dissection has shown that several signal factors are also required for the *Verticillium* wilt resistance mediated by tomato Ve1, including enhanced disease susceptibility 1 (EDS1), non-race-specific disease resistance 1 (NDR1), BRI1-associated kinase 1 (BAK1), and mitogen-activated protein kinase kinase 2 (MKK2) (Fradin et al., 2009).

In cotton plants, *Verticillium* wilt is difficult to control because of the lack of resistance germplasm resources. Recently, several *Ve1* gene homologs have been isolated from island cotton *Gossypium barbadense*, including *GbVe, GbVe1*, and *Gbvdr3*, and have been shown to confer *Verticillium* wilt resistance by virus-induced gene silencing (VIGS) in cotton or overexpression in *Arabidopsis* (Zhang et al., 2011, 2012; Chen T. et al., 2015; Yang et al., 2015). Here, two *RLP* gene homologs, *GbaVd1* and *GbaVd2*, were cloned from the *Verticillium* wilt-resistant cultivar *G. barbadense* cv. Hai7124. *GbaVd1* and *GbaVd2* encode typical RLPs, conditionaly enhanced *Verticillium* wilt resistance susceptibility against *V. dahliae* through VIGS in cotton plants, and enhanced *Verticillium* wilt resistance after interfamily transfer into *Arabidopsis*. Finally, the *GbaVd1*- and *GbaVd2*-mediated resistance signal transduction mechanisms and activation of defense responses were investigated by microarray analysis in transgenic *Arabidopsis* plants.

## Materials and methods

### Plant material, fungal culture, and inoculation

Seedlings of the resistant cultivar *G. barbadense* cv. Hai7124 and six cotton species (*Gossypium anomalum, Gossypium trilobum, Gossypium aridum, Gossypium davidsonii, Gossypium thurberi*, and *Gossypium hirsutum*) were planted in a 12-cm pot with sterilized soil at 28°C with a 14/10 light-dark photoperiod for 2 weeks, as previously described (Chen and Dai, 2010). The highly virulent *V. dahliae* strain Vd991 was cultured on potato dextrose agar (PDA) medium at 25°C for 10 days, the conidia were then washed with sterile water and the concentration was adjusted to 2 × 10^7^ conidia/mL. Six 2-week-old seedlings were each inoculated with 5 mL of conidial suspension by a soil drenching method. To prepare the samples for detection of *GbaVd1* and *GbaVd2* expression, the root tissues were collected 2, 6, 12, 24, and 48 h after inoculation.

### Gene cloning

RNA samples from inoculated plants were used for gene cloning. The RNA was extracted by using the cetyltrimethylammonium bromide (CTAB) method (Bekesiova et al., 1999). After tracing DNA in the RNA samples was removed by treatment with DNase I, cDNA was synthesized using a RevertAid™ First Strand cDNA Synthesis Kit from MBI (Fermentas, Glen Burnie, Maryland, USA). The primers specific to the cotton *RLP* gene homologs, *GbaVd1* and *GbaVd2* (cloned in our lab by homology-based cloning, the accession numbers is GU299533 and GU299534, respectively), were designed and used to amplify the cDNA samples. Primers used for gene cloning are listed in Table S1.

### Bioinformatics analysis of *Gbavd1* and *Gbavd2*

The open reading frames (ORFs) of *GbaVd1* and *GbaVd2* were determined by using ORF Finder (NCBI), and the protein sequences were deduced on the basis of gene sequences. The identities between GbaVd1 and GbaVd2 were determined by using the Blast program (http://www.ncbi.nlm.nih.gov/Blast.cgi) and Vector NTI software (Lu and Moriyama, 2004). Signal peptides and membrane-spanning structures were predicted by using SignalP 4.0 (Petersen et al., 2011) and TMHMM 2.0 (Sonnhammer et al., 1998), respectively. The primary protein structure was determined by protein sequence alignment with known RLPs, including *Cf-2.2* (U42445), *Cf-5* (AF053993), *Cf-4* (AJ002235), *Cf-9* (AJ002236), *HcrVf1* (AJ297739), *HcrVf2* (AJ297740), *HcrVf3* (AJ297741), *LeEix1* (AY359965), *LeEix2* (AY359966), *Ve1* (AF365929), and *Ve2* (AF272366). ClustalX 1.83 software was used for multiple sequence alignment (Thompson et al., 1997), and data were exported with Boxshade 3.21 (http://www.ch.embnet.org/software/BOX_form.html, written by K. Hofmann and M. Baron). Phylogenetic trees were constructed in Mega 6.0 with the Maximum Parsimony method, using the Jones-Taylor-Thornton (JTT) model and performing 1000 bootstrap replicates (Tamura et al., 2013).

### VIGS in cotton and detection of *Verticillium* wilt resistance

For the VIGS assays, *GbaVd1* and *GbaVd2* were integrated into a vector and introduced into *A. tumefaciens* GV3101. *Agrobacterium* the strains harboring the pTRV2-GbaVd1/GbaVd2 plasmid combined with strains harboring the pTRV1 vector were mixed in a 1:1 ratio and co-infiltrated into the cotyledon leaves of 2-week-old cotton plants. The effectiveness of the VIGS assay was evaluated by using the control cotton *CLA1* gene as previously described (Gao and Shan, 2013). Approximately 14 days after the VIGS procedure, a visible phenotype of white-colored leaves were observed in plants in which the *CLA1* gene had been targeted by VIGS, and all of the plantlets were subjected to *V. dahliae* inoculation with 5 mL of conidial suspension (2 × 10^7^ conidia/mL). The *Verticillium* wilt symptoms were investigated 3 weeks after inoculation. Fungal biomass in cotton were determined using a method described previously (Santhanam et al., 2013). qPCR was performed using a qPCR SYBR premix Ex Taq II kit (TaKaRa, Japan) with primers specific to the cotton *18S* gene and *V. dahliae* elongation factor 1-α (*EF-1*α) (Table S1).

### Arabidopsis transformation and evaluation of transgenic disease resistance

According to the sequences of *GbaVd1* and *GbaVd2*, the ORF fragments were amplified with primers containing *Bam*H I and *Bst*E II enzyme sites. The fragments of *GbaVd1* and *GbaVd2* were integrated into the binary vector pCAMBIA1303 under control of the cauliflower mosaic virus (CaMV) 35S promoter. The recombinant vectors were transferred into *A. tumefaciens* strain LBA4404, and this was followed by genetic transformation of *A. thaliana* ecotype *Col-0* via the floral dip transformation method (Clough and Bent, 1998). Homozygous transgenic *Arabidopsis* (T_3_) plants were screened and used for this study. Transgenic plants were selected on MS medium containing 50 mg/L hygromycin and were identified by RT-PCR. The RT-PCR conditions consisted of an initial 94°C denaturation step for 10 min, which was followed by 35 cycles of 94°C for 30 s, 55°C for 30 s, and 72°C for 30 s, and ubiquitin 4 (*Ubi4*, NM_122069.3) was used as a control. Two random transgenic plant lines carrying *GbaVd1* and *GbaVd2* were selected for the *Verticillium* wilt resistance assay. The inoculation method was performed as in cotton. In brief, 4-week-old seedlings were inoculated with 5 mL of conidial suspension (5 × 10^6^ conidia/ml). The inflorescence height was detected 4 weeks after inoculation. Likewise, the development of fungal biomass in transgenic plants was detected by qPCR, with primers to RNA-binding family proteins in *Arabidopsis* (Table S1).

### Microarray analysis

Microarray analysis was performed using *Arabidopsis* Gene Expression Microarray Version 4.0 (43,803 probes) (Agilent Technologies, USA). Three independent transgenic lines of *GbaVd1* and *GbaVd2* overexpression were used for microarray analysis. Two-week-old seedling transgenic plants without inoculation were collected for RNA extraction, and the wild type (Col-0) was used as a control. Total RNA was extracted from the root and labeled with Cy-3 using a Low RNA Input Linear Amplification/Labeling Kit, One-color (Agilent Technologies), hybridized, and washed according to the manufacturer's instructions. Hybridized microarrays were then scanned with an Agilent Microarray Scanner (Agilent Technologies). Statistical data extraction processes were performed on three biological replicates for each treatment according to the instructions (Agilent, GeneSpring GX9 software package). Fold changes in expression levels in response to each transgenic plant were compared with those of the respective wild-type, *P*-values were adjusted for multiple comparisons using the Bonferroni correction. Sequence homologies of the deduced amino acids were determined using the Arabidopsis Information Resource (TAIR) database (http://www.Arabidopsis.org/). Functional annotation of GO and metabolic pathways were performed using the GeneSpring GX9 package (Agilent Technologies, USA), and statistically for overrepresented GO terms. The significance of GO catalog for differentially expressed genes were identified using a *Fisher's* Exact Test (filtered with *FDR* ≤ 0.05) as previously described (Chen et al., 2016).

### Expression of *Gbavd1* and *Gbavd2*

The cDNA samples were used for qRT-PCR analysis with SYBR Green (Invitrogen, Carlsbad, California, USA), and the PCR was performed using a thermocycler (ABI PRISM 7500, Applied Biosystems) with the following program: 94°C for 30 s, 55°C for 30 s, and 72°C for 30 s for 40 cycles. The cotton *18S* gene was used as an endogenous control. Relative transcript levels of *GbaVd1* and *GbaVd2* were determined using the 2^−ΔΔCT^ method (Livak and Schmittgen, 2001), with three independent determinations.

### Lignin histochemical staining

Four-week-old seedlings of *Arabidopsis* transgenic lines were inoculated with 5 mL of conidial suspension (5 × 10^6^ conidia/ml). The internode of shoot tissue between the second and third nodes was used for cell wall reinforcement detection at 4 weeks after inoculation. The samples were stained with phloroglucinol solution (3% w/v phloroglucinol in 95% ethanol) for 2 min after slice treatment and were then immersed in concentrated HCl for a few seconds and covered with glycerol (Nakano and Meshitsuka, 1992). The tissue slices were observed with a Leitz Diaplan light microscope and photographed immediately.

## Results

### Isolation of *Gbavd1* and *Gbavd2* from cotton

**Figure 1:**
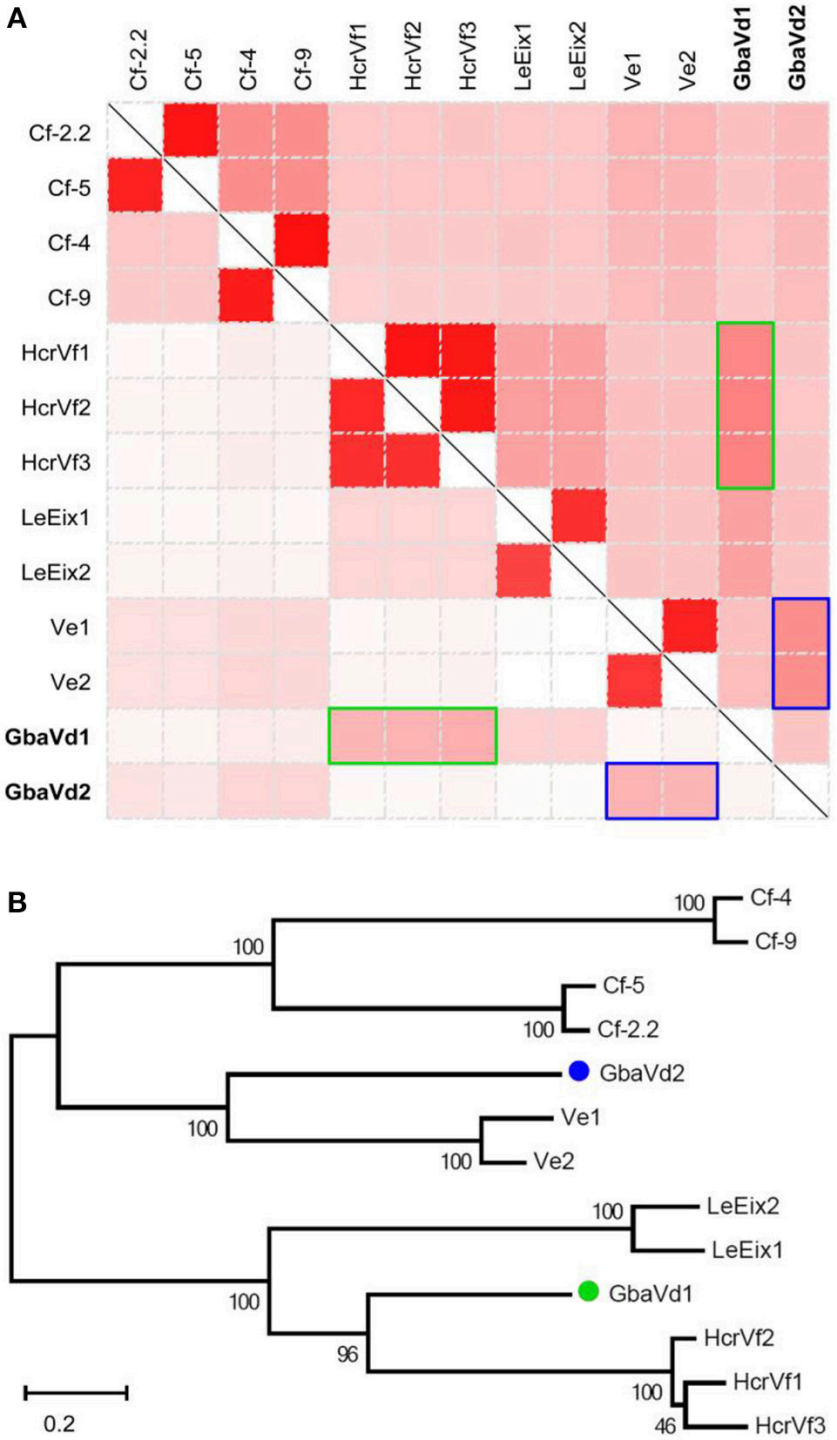
**Analysis of ***GbaVd1*** and ***GbaVd2*** sequence characteristics. (A)** Homology analysis of *GbaVd1* and *GbaVd2* with known RLPs. The upper-right and bottom-left represent identical nucleotide and encoding protein sequences among RLPs, respectively. The colors, from white to red, represent the percentage identities, from 0 to 100%. The green and blue rectangles represent the best hits of RLPs with *GbaVd1* and *GbaVd2*, respectively. **(B)** Phylogenetic tree of *GbaVd1* and *GbaVd2* with homologs from other plant species. The analysis was performed using the package MEGA6 with the Maximum Parsimony method.

The full-length of *GbaVd1* and *GbaVd2* were cloned from the cDNA sample of *G. barbadense* (cv. Hai7124) inoculated with *V. dahliae* by PCR (Figure S1A). Sequences analysis showed that *GbaVd1* and *GbaVd2* are 3,262 and 3,466 bp in length, respectively, without any introns, and each has a single open reading frame (ORF) of 1,020 or 1,082 amino acids (Figure S1B), respectively. BlastP analysis revealed that both contain an LRR ribonuclease inhibitor domain, a potential signal peptide and a transmembrane region (Figure S2), and both were predicted to be located in the plasma membrane (the *testk* used for *kNN* was 14, and the Nearest Neighbors numbers of *GbaVd1* and *GbaVd2* related to the plasma membrane were 9 and 8, respectively). Sequence alignment indicated that the protein-coding sequences of *GbaVd1* and *GbaVd2* are similar to those of known plant RLP genes, with *GbaVd2* showing the highest identities to *Ve* genes, but with *GbaVd1* displaying the highest identities to *HcrVf* genes (Figure 1A). Likewise, the alignment of encoded protein sequences also confirmed that *GbaVd1* and *GbaVd2* are most homologous to *Ve* and *HcrVf* genes (Figure 1A), respectively. Sequence alignment showed that GbaVd2 is a homolog to Ve-like proteins from *G. barbadense*, and is nearly identical to the previously cloned and published Gbvdr5 (Yang et al., 2015) with only one conservative amino acid change (Figure S3). GbaVd1 protein sequence showed high identity to a RLP from cotton (Figure S4). In addition, six allelic variants of the *GbaVd1* and *GbaVd2* were isolated from six different cotton species, and two of them are susceptible to *V. dahliae*. A DNA sequence alignment showed a total of 125 and 264 single nucleotide polymorphisms (SNPs) among the allelic variants compared with the reference sequence of *GbaVd1* and *GbaVd2* (Figures S5A,C), resulting in 74 and 177 amino acid changes (Figures S5B,D), respectively. However, the association of the amino acid substitutions with the Verticillium wilt resistance of cotton species was not found among these allelic variants (Figures S5B,D). Phylogenetic analysis showed a close relationship between *GbaVd1* and *HcrVfs*, which clustered into the same branch, and between *GbaVd2* and *Ve* genes (Figure 1B). These results suggested that both *GbaVd1* and *GbaVd2* display homology to the known RLPs but also show sequence divergence when compared with each other.

### *Gbavd1* and *Gbavd2* encode typical RLPs

**Figure 2:**
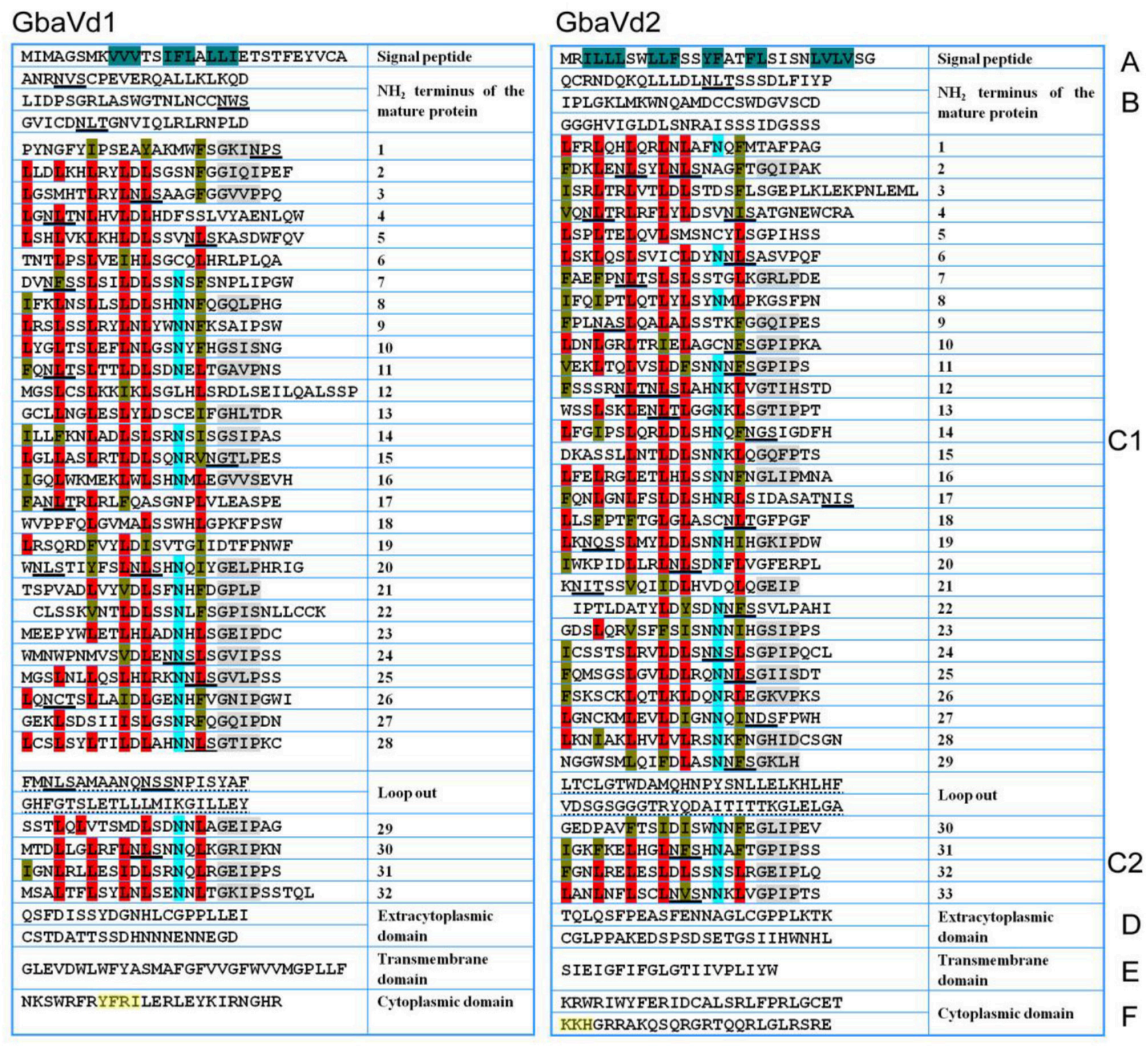
*****GbaVd1*** and ***GbaVd2*** encoded typical RLPs**. The primary structures were divided into domains **(A–F)**. Domain **(A)** is the putative signal peptide, residues in dark green are hydrophobic amino acids; domain **(B)** is the putative N-terminus of the mature protein; domain **(C)** (divided into **C1,C2**) is the LRR domain, in which conserved leucine residues (L, red) are often replaced by isoleucine (I), phenylalanine (F), or valine (V) (brown), conserved N-termini are highlighted in light blue, and the conserved GxIP motif is depicted in gray; domain **(D)** is a negatively charged extracytoplasmic peptide with neutral and acidic amino acids; domain **(E)** is the transmembrane-associated domain; domain **(F)** is the small cytoplasmic peptide with neutral and basic amino acids, with the endocytosis signal motifs highlighted in yellow. Putative *N*-glycosylation sites Nx(S/T) present in domains **(B,C)** are underlined with black lines.

Although the sequence identities between *GbaVd1* or *GbaVd2* and known RLPs were relatively low (Figure 1A), the alignment results showed that the amino acid residues involved in the LRR structure are conserved (Figure S6). Sequence alignments between GbaVd1 or GbaVd2 and known RLPs indicated that both contain six conserved domains, Domains A through F (Figure 2). Domain A consists of the signal peptide and its cleavage site at the N-terminus of the protein. Domain B belongs to the N-terminus of the mature protein. Domain C contains LRR structures, with 32 and 33 LRRs in GbaVd1 and GbaVd2, respectively. Domain C is usually divided into two sections, domains C1 and C2, which are linked by a small peptide, and the number of LRRs (four) in domain C2 is identical between GbaVd1 and GbaVd2. Domains B and C also contain 22 and 26 putative *N*-glycosylation sites Nx(S/T) in GbaVd1 and GbaVd2, respectively. Domain D is a negatively charged extracytoplasmic domain predicted to play a role in orienting and anchoring the protein to the cell membrane and links domain C with the transmembrane structure of domain E. Domain F is a small cytoplasmic peptide with positively charged residues and is linked to domain E. In addition, the cytoplasmic domain of GbaVd1 possesses a YXXø signal sequence (YFRI), and GbaVd2 contains a KKX motif (KKH), both of which probably participate in the endocytosis process (Figure 2). A sequence similarity search using GbaVd1 showed high identity to a protein annotated as a LRR receptor-like serine/threonine-protein kinase FLS2 from cotton (Figure S4). However, there is no kinase domain in the small cytoplasmic region, suggesting improper annotation of the FLS2 homolog and that GbaVd1 encodes a RLP (not an RLK). Therefore, similar to the known RLPs, *GbaVd1* and *GbaVd2* encode extracytoplasmic glycoproteins in which the majority of the extracytoplasmic domain consists of multiple LRRs.

### Silencing *Gbavd1* and *Gbavd2* compromise resistance to *V. dahliae* in cotton

**Figure 3:**
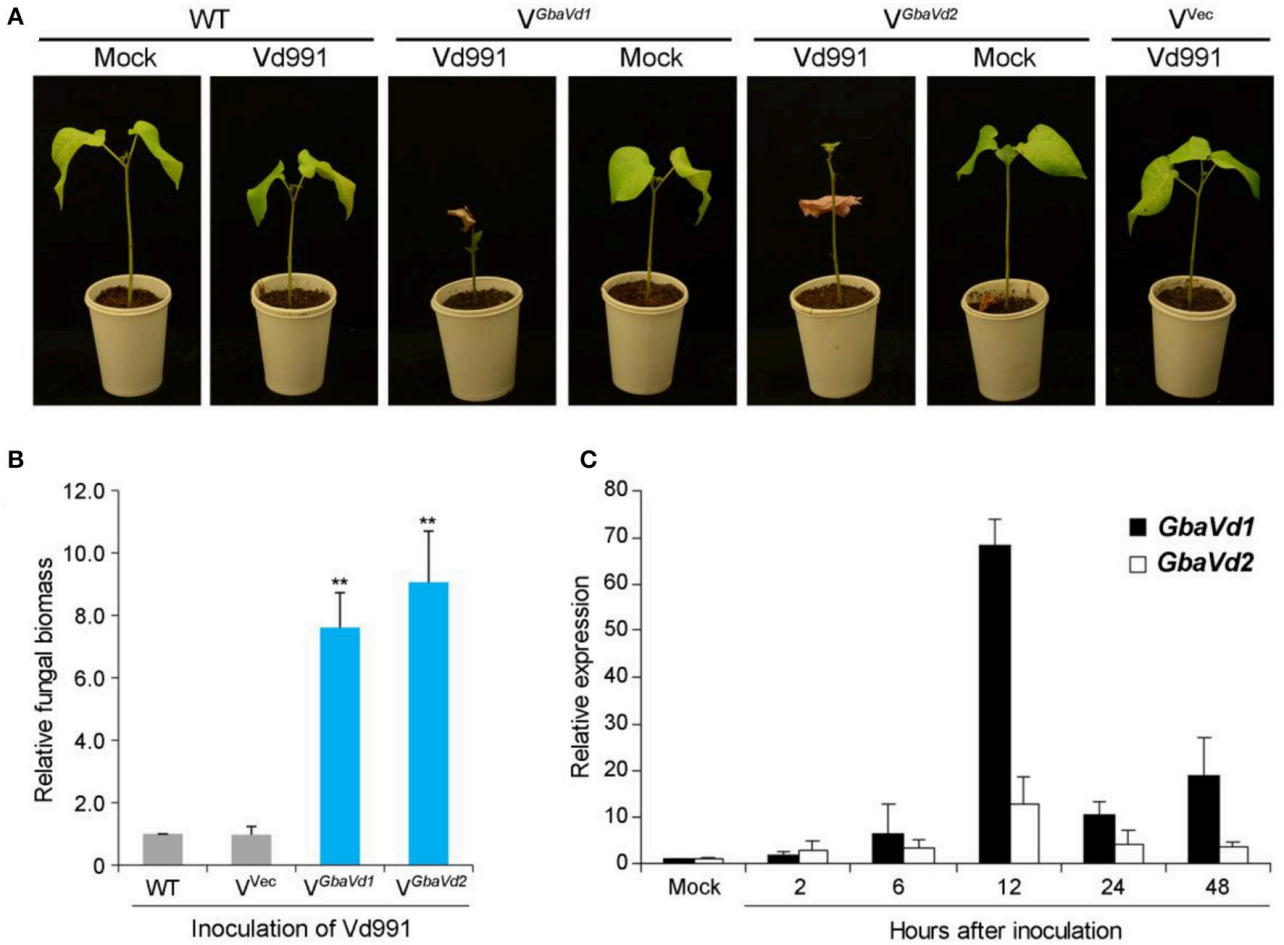
**The ***GbaVd1*** and ***GbaVd2*** genes play essential roles in ***Verticillium*** wilt resistance in cotton. (A)** Determination of *Verticillium* wilt resistance in *GbaVd1*- or *GbaVd2*-silenced cotton plants. Approximately 14 days after the VIGS procedure in the resistant cotton *G. barbadense* cv. Hai7124, the *GbaVd1*- or *GbaVd2*-silenced plants were inoculated with 5 mL of conidial suspension (2 × 10^7^ conidia/mL) from *V. dahliae* strain Vd991. The *Verticillium* wilt phenotypes were determined and photographed 3 weeks after inoculation. WT, the wild type of *G. barbadense* cv. Hai7124; Mock, inoculation with sterile water; V^GbaVd1^/V^GbaVd2^, the *GbaVd1*- or *GbaVd2*-silenced plants, respectively; V^vec^, VIGS positive control of infiltration with the empty vector pTRV2. **(B)** Real-time PCR quantification of fungal biomass in *GbaVd1*- or *GbaVd2*-silenced plants. Error bars represent standard errors, ^**^significant differences (*P* ≤ 0.01), according to unpaired Student's *t*-test. **(C)** Expression patterns of *GbaVd1* or *GbaVd2* in cotton after inoculation with *V. dahliae*. Relative gene quantifications over time were calculated using the comparative threshold (2^−ΔΔCT^) method, and three independent biological replicates were analyzed. Error bars represent standard errors.

To identify the roles of *GbaVd1* and *GbaVd2* in *Verticillium* wilt resistance, tobacco rattle virus (TRV)-based virus-induced gene silencing was used in the island cotton cv. Hai7124. The cotton gene cloroplastos alterados 1 (*CLA1*), which is essential for chloroplast development, was used as a positive control to monitor VIGS efficiency. Plants in which the *GbaVd1* or *GbaVd2* expression was silenced by TRV-based VIGS were challenged with *V. dahliae* after all of the *CLA1*-silenced cotton plants presented an albino phenotype on their newly emerged leaves. At 21 days post-inoculation with the *V. dahliae* strain V991, plants treated with TRV-based VIGS of *GbaVd1* or *GbaVd2* but not the wild-type plants presented clear *Verticillium* wilt symptoms of leaf chlorosis, including withering and dwarfing (Figure 3A), thus indicating that the resistant cotton cv. Hai7124 displayed compromised resistance to *V. dahliae* after silencing of *GbaVd1* or *GbaVd2* by VIGS. Real-time quantitative polymerase chain reaction (qPCR) quantification of gene expression related to fungal biomass development demonstrated significantly increased *V. dahliae* strain Vd991 propagation in *GbaVd1*- or *GbaVd2*-silenced plants compared with the wild-type and empty vector-treated plants (Figure 3B). Moreover, *GbaVd1* and *GbaVd2* transcript accumulation was quickly and strongly induced 2–48 h after inoculation with *V. dahliae* and reached the highest expression levels (>10-fold change) 12 h after inoculation (Figure 3C). Thus, it can be concluded that the functions of *GbaVd1* and *GbaVd2* are closely associated with *Verticillium* wilt resistance in cotton.

### Heterologous overexpression of *Gbavd1* and *Gbavd2* confer *Verticillium* wilt resistance in *Arabidopsis*

**Figure 4:**
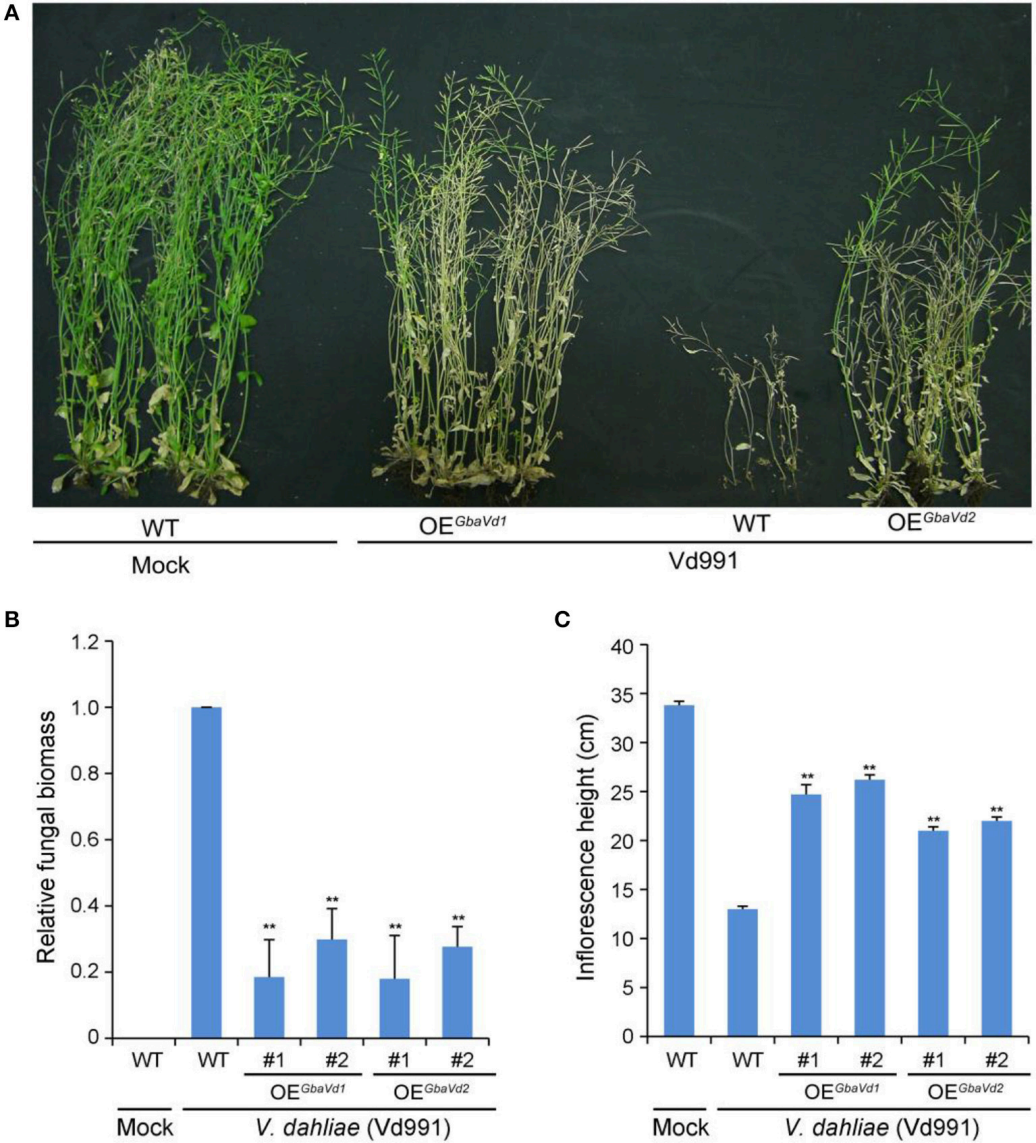
*****GbaVd1*** and ***GbaVd2*** conferred ***Verticillium*** wilt resistance in transgenic ***Arabidopsis***. (A)** Identification of *Verticillium* wilt resistance after interfamily transfer of *GbaVd1* and *GbaVd2* in *Arabidopsis*. Four-week-old seedlings of homozygote transgenic *Arabidopsis* (T_3_) were inoculated with 5 mL of conidial suspension (5 × 10^6^ conidia/mL). The *Verticillium* wilt phenotypes were determined and photographed 4weeks after inoculation. WT, the wild-type *Col-0*; Mock, inoculation with sterile water; OE^GbaVd1^/OE^GbaVd2^, transgenic plants overexpressing *GbaVd1* or *GbaVd2*, respectively. **(B)** Real-time PCR quantification of fungal biomass in *GbaVd1* or *GbaVd2* transgenic plants. Error bars represent standard errors, ^**^significant differences (*P* ≤ 0.01), according to unpaired Student's *t*-test. **(C)** Investigation of the inflorescence height after inoculation with *V. dahliae*. Error bars represent standard errors, ^**^significant differences (*P* ≤ 0.01), according to unpaired Student's *t*-test.

To identify the *Verticillium* wilt resistance functions of *GbaVd1* and *GbaVd2*, both were interfamily-transferred into the *Arabidopsis* genome by using the *Agrobacterium tumefaciens*-mediated transformation method. Ten *GbaVd1* and nine *GbaVd2* independent *Arabidopsis* transgenic lines were obtained by hygromycin-resistance selection and verified by reverse transcription PCR (RT-PCR), and the integrated genes were successfully expressed, except in one *GbaVd2 Arabidopsis* transgenic line (Figure S7). Two random transgenic plant lines of *GbaVd1* and *GbaVd2* were selected for *Verticillium* wilt resistance identification with the root dip method and were assessed for *Verticillium* wilt resistance by evaluation of the extent of leaf chlorosis and inflorescence heights. Compared with the wild-type *Col-0* ecotype, overexpressed *GbaVd1* or *GbaVd2* in *Arabidopsis* improved the resistance of the transgenic plants to *V. dahliae* strain Vd991, as indicated by significant decreases in leaf chlorosis, withering and dwarfing (Figure 4A). More importantly, unlike the wild-type *Arabidopsis*, which lost the capacity to set seed, the transgenic plants presented with a normal seed set 4 weeks after inoculation with *V. dahliae* (Figure 4A), a result strongly suggesting that overexpression of *GbaVd1* or *GbaVd2* conferred *Verticillium* wilt resistance to *Arabidopsis*. Further examination by qPCR confirmed that the *V. dahliae* significantly developed less fungal biomass in *GbaVd1* or *GbaVd2* transgenic plants than in the wild-type plants (Figure 4B). In addition, plant dwarfing, a typical *Verticillium* wilt symptom, was also significantly alleviated in transgenic lines after inoculation with *V. dahliae* (Figure 4C). Together, these results strongly suggested that *GbaVd1* and *GbaVd2* function as resistance genes and that interfamily transfer of these genes confers *Verticillium* wilt resistance in *Arabidopsis*.

### Microarray analysis of gene expression profiles in the *Gbavd1* and *Gbavd2* transgenic lines

**Figure 5:**
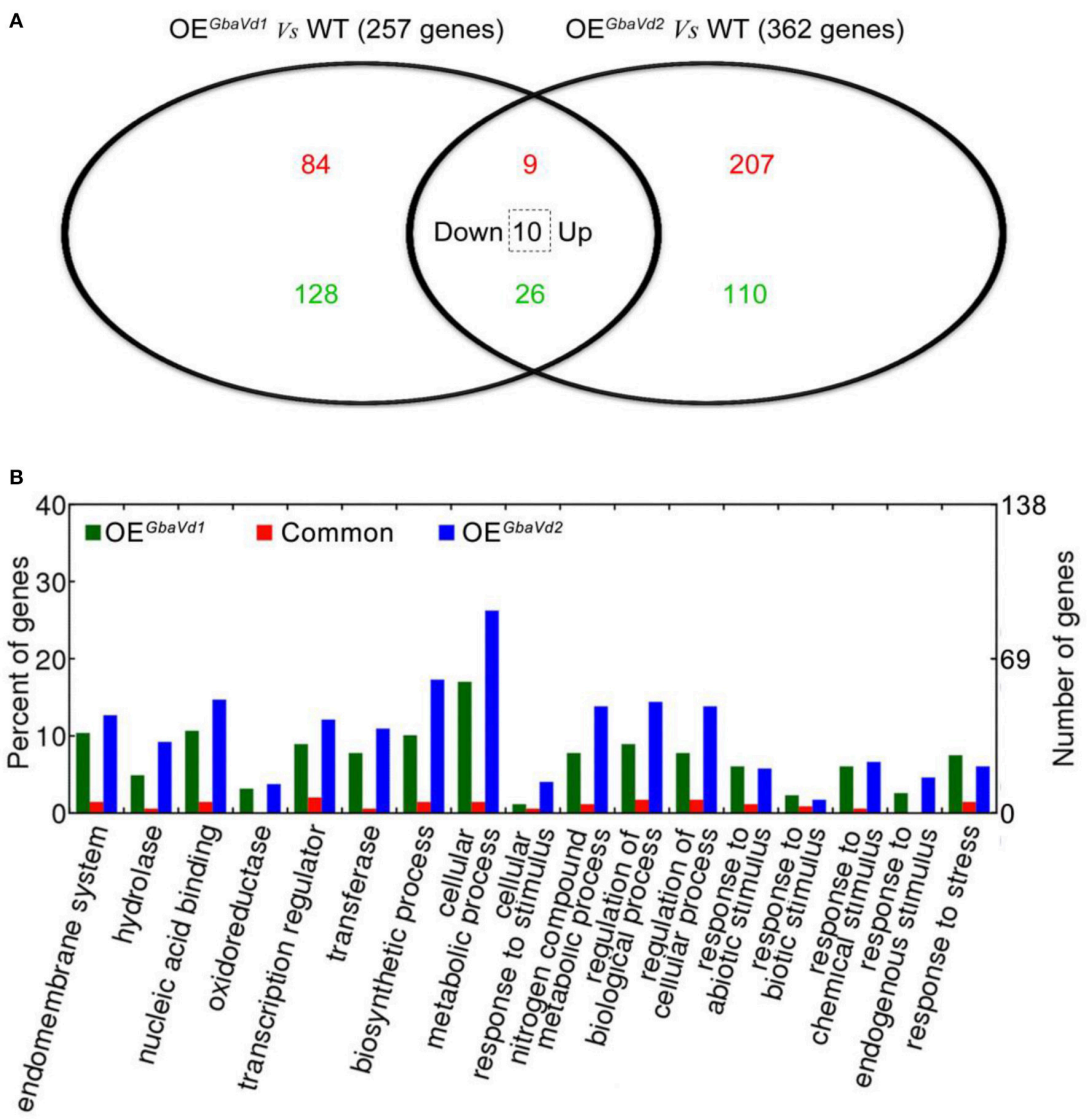
**Microarray analysis of differentially expressed genes in ***GbaVd1*** and ***GbaVd2*** transgenic lines. (A)** The Venn diagram represents the differentially expressed genes between the transgenic and wild-type (WT) lines under normal conditions. The red numbers represent the up-regulated genes, the green numbers represent the down-regulated genes, and the number in the dotted box represents genes down-regulated in the *GbaVd1* transgenic line but up-regulated in the *GbaVd2* transgenic line. **(B)** GO analysis of differentially expressed genes in *GbaVd1* and *GbaVd2* transgenic lines.

To identify the defense responses mediated by *GbaVd1* and *GbaVd2*, the transcriptome of transgenic lines were assayed by using an Agilent 44 K custom oligo microarray system with three independent biological replicates. In total, 4,153 and 4,484 probes were detected in *GbaVd1* and *GbaVd2* transgenic lines (Supplementary Data S1), respectively. Of these detected genes, 257 (93 genes up-regulated and 164 genes down-regulated) showed differential expression in the *GbaVd1* transgenic line (fold-change ≥1.5, *p* < 0.01), and 362 (226 genes up-regulated and 136 genes down-regulated) were differentially expressed in the *GbaVd2* transgenic line (fold-change ≥1.5, *p* < 0.01) (Figure 5A; Table S2). A further comparison of the two differentially expressed gene sets revealed 45 common genes between the *GbaVd1* and *GbaVd2* transgenic lines, of which 35 displayed the same regulation patterns (nine genes up-regulated and 26 genes down-regulated), and the other 10 of which showed down-regulation in the *GbaVd1* transgenic line but up-regulation in the *GbaVd2* transgenic line (Figure 5A; Table S2). These results indicated that the regulation patterns mediated by *GbaVd1* and *GbaVd2* display some of the same aspects, but that each has unique characteristics. Gene ontology (GO) analysis indicated that subsets of the differentially expressed genes activated by *GbaVd1* and *GbaVd2* were involved in the categories cellular metabolic process (statistically in overrepresented, filtered with *FDR* ≤ 0.05), regulation of cellular process, response to stress, and transcription regulation (Figure 5B). These results suggested that *GbaVd1* and *GbaVd2* have similar functions in mediating the defense response after overexpression in *Arabidopsis*.

### Overexpression of *Gbavd1* and *Gbavd2* altere the expression of defense-related genes in *Arabidopsis*

**Figure 6:**
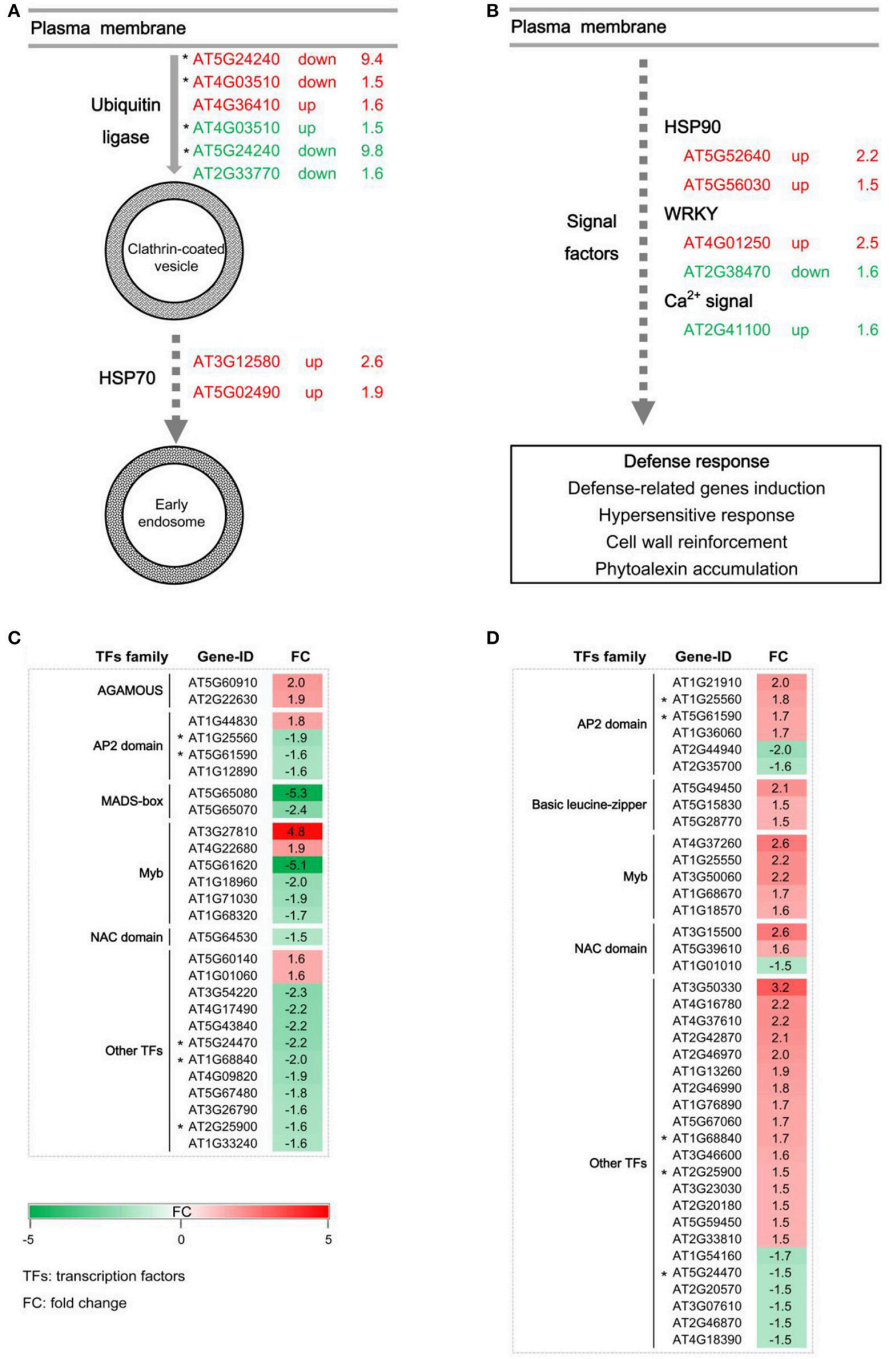
**Pathway enrichment analysis of differentially expressed genes in ***GbaVd1*** and ***GbaVd2*** transgenic plants. (A)** Differentially expressed genes involved in the endocytosis process. The differentially expressed genes associated with the *GbaVd1* and *GbaVd2* transgenic plants are shown in red and green, respectively. **(B)** Differentially expressed genes involved in signal transduction. **(C,D)** Activation of genes encoding transcription factors after *GbaVd1* **(C)** or *GbaVd2* **(D)** overexpression in *Arabidopsis*. The red and green blocks represent the up- and down-regulated patterns, respectively, and genes with asterisks represent the common activation genes between the *GbaVd1* and *GbaVd2* transgenic lines.

Generally, the endocytosis process and signal transduction are necessary for the disease resistance mediated by RLPs. *GbaVd1* and *GbaVd2* contain a conserved motif that is probably involved in the endocytosis process (Figure 2). As expected, several genes associated with endocytosis were differentially expressed after *GbaVd1* and *GbaVd2* overexpression in *Arabidopsis*, including genes encoding ubiquitin ligase family proteins and heat shock protein 70 (HSP70) (Figure 6A; Table S3). Specifically, there are three genes encoding ubiquitin ligase family proteins, two of which were commonly activated in the *GbaVd1* and *GbaVd2* transgenic lines. These proteins facilitate formation of clathrin-coated vesicles during endocytosis (Figure 6A; Table S3). In addition, two *HSP70* genes (AT3G12580 and AT5G02490) involved in the formation of early endosomes were also differentially expressed in the *GbaVd1* transgenic line (Figure 6A; Table S3). These results suggested that the endocytosis process associated with plant-pathogen interactions was affected after *GbaVd1* and *GbaVd2* overexpression in *Arabidopsis*. In addition, several genes encoding signal factors involved in plant-pathogen interactions were also differentially expressed in the *GbaVd1* and *GbaVd2* transgenic lines (Figure 6B; Table S3). In the *GbaVd1* transgenic line, the genes encoding two heat shock protein 90 (HSP90) isoforms and the transcription factor WRKY33 were up-regulated, but two other genes encoding calmodulin-like (CaM/CML) protein 12 and the transcription factor WRKY22 were activated in the *GbaVd2* transgenic line (Figure 6B). Moreover, 27 and 39 genes encoding transcription factors were significantly differentially expressed after overexpression of *GbaVd1* and *GbaVd2*, respectively; these included members of the AP2 domain-containing protein family and the myeloblastosis (Myb) family (Figures 6C,D; Table S3). However, most transcription factor genes were specifically differentially expressed in either the *GbaVd1* or *GbaVd2* transgenic lines, except for five common genes (Figures 6C,D; Table S3). Together, these results indicated that the interfamily transfer of cotton *GbaVd1* and *GbaVd2* provided the capacity to differentially expressed defense response-related genes and mediate *Verticillium* wilt resistance in *Arabidopsis*.

### Overexpression of *Gbavd1* and *Gbavd2* result in cell wall reinforcement in *Arabidopsis*

**Figure 7:**
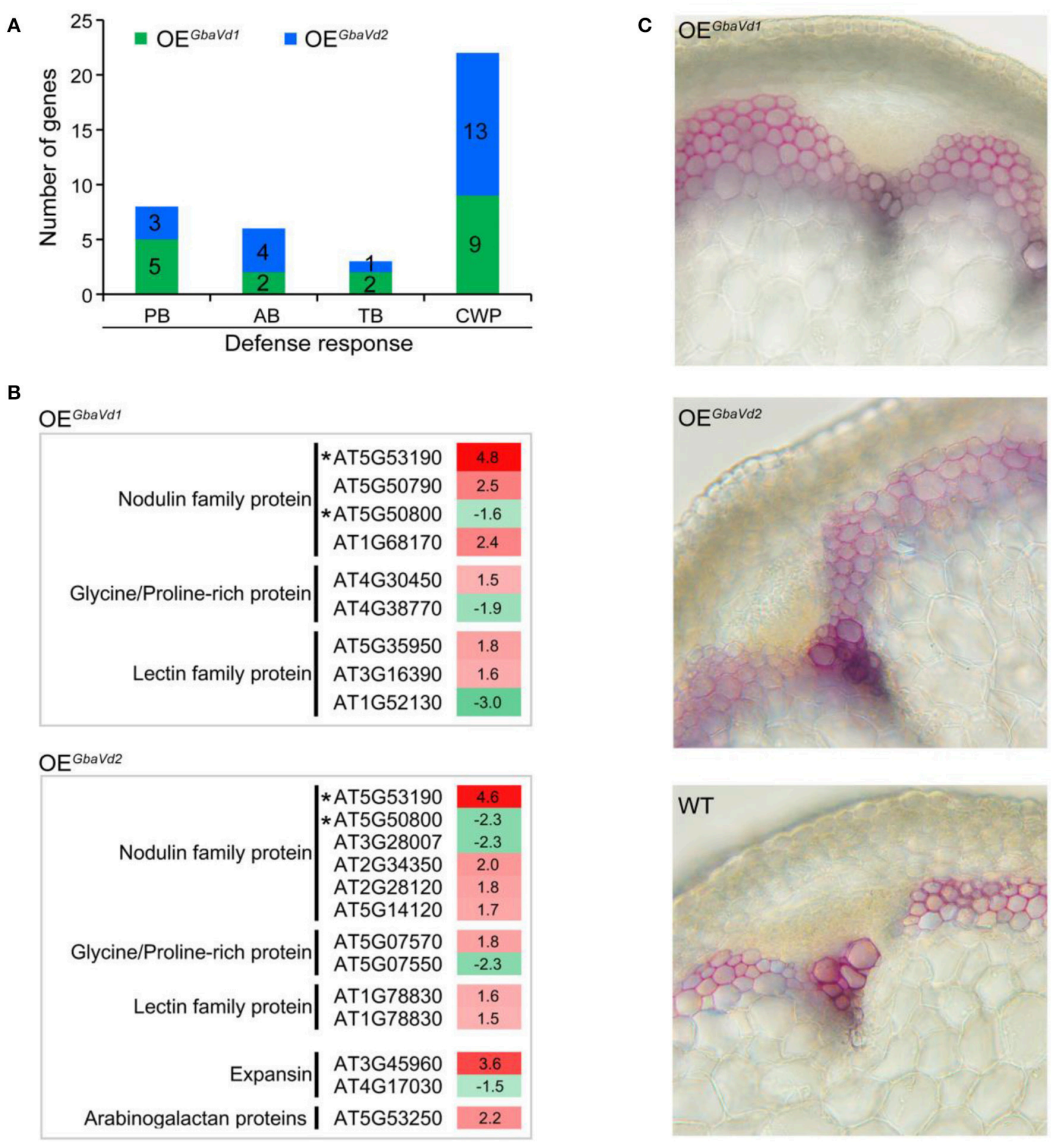
**Analysis of the activation of defense responses in ***GbaVd1*** and ***GbaVd2*** transgenic lines. (A)** Enrichment analysis pathway of differentially expressed genes participating in the defense response. PB, phenylpropanoid biosynthesis; AB, alkaloid biosynthesis; TB, terpenoid biosynthesis; CWP, cell wall-related protein. **(B)** List of cell wall-related proteins activated after *GbaVd1* or *GbaVd2* overexpression in *Arabidopsis*. Genes with asterisks represent the common activation genes between *GbaVd1* and *GbaVd2* transgenic lines. **(C)** Detection of the lignification of *GbaVd1* and *GbaVd2* transgenic lines. The transgenic plant shoot tissues between the second and third nodes were collected 4 weeks after inoculation with *V. dahliae* strain Vd991. The lignification phenotypes were determined and photographed after staining via the phloroglucinol-HCl method.

Plants usually enhance disease resistance by activation of defense responses that are mediated by the plant-pathogen interaction pathway (PATHWAY: map04626, KEGG database). In this study, the plant-pathogen interaction pathway was significantly activated after *GbaVd1* or *GbaVd2* overexpression in *Arabidopsis*, thus resulting in the induction of defense responses, including the hypersensitive response, phytoalexin accumulation, and cell wall reinforcement (Figures 6A,B). Microarray analysis confirmed that several differentially expressed genes that were significantly enriched were involved in the phytoalexin (phenylpropanoids, alkaloids, terpenoids, etc.) biosynthesis pathway or encode cell wall-related proteins (Figure 7A; Table S4).

Functional analysis of differentially expressed genes revealed that 9 and 13 genes encoding cell wall-related proteins were differentially expressed by overexpression of *GbaVd1* or *GbaVd2*, respectively, in *Arabidopsis* (Figure 7A). Of these genes, the most encoded were nodulin family proteins, glycine/proline-rich proteins, and lectin family proteins, and were mainly up-regulated in transgenic lines (Figure 7B). Specifically, for the nodulin family, four and six genes were significantly activated in the *GbaVd1* and *GbaVd2* transgenic lines, respectively (Figure 7B). Moreover, the identification of cell wall structures confirmed that the degree of lignification was significantly enhanced by the transcription of genes encoding cell wall-related proteins (Figure 7C), thus indicating that the cell wall is reinforced after *GbaVd1* or *GbaVd2* overexpression in *Arabidopsis*. In addition, of these cell wall-related genes, only two genes were commonly regulated between the *GbaVd1* and *GbaVd2* transgenic lines (Figure 7B), thus suggesting that the cell wall-related proteins were differentially activated in the two transgenic lines.

### *Gbavd1* and *Gbavd2* differentially regulate defense responses, enhancing *Verticillium* wilt resistance

**Figure 8:**
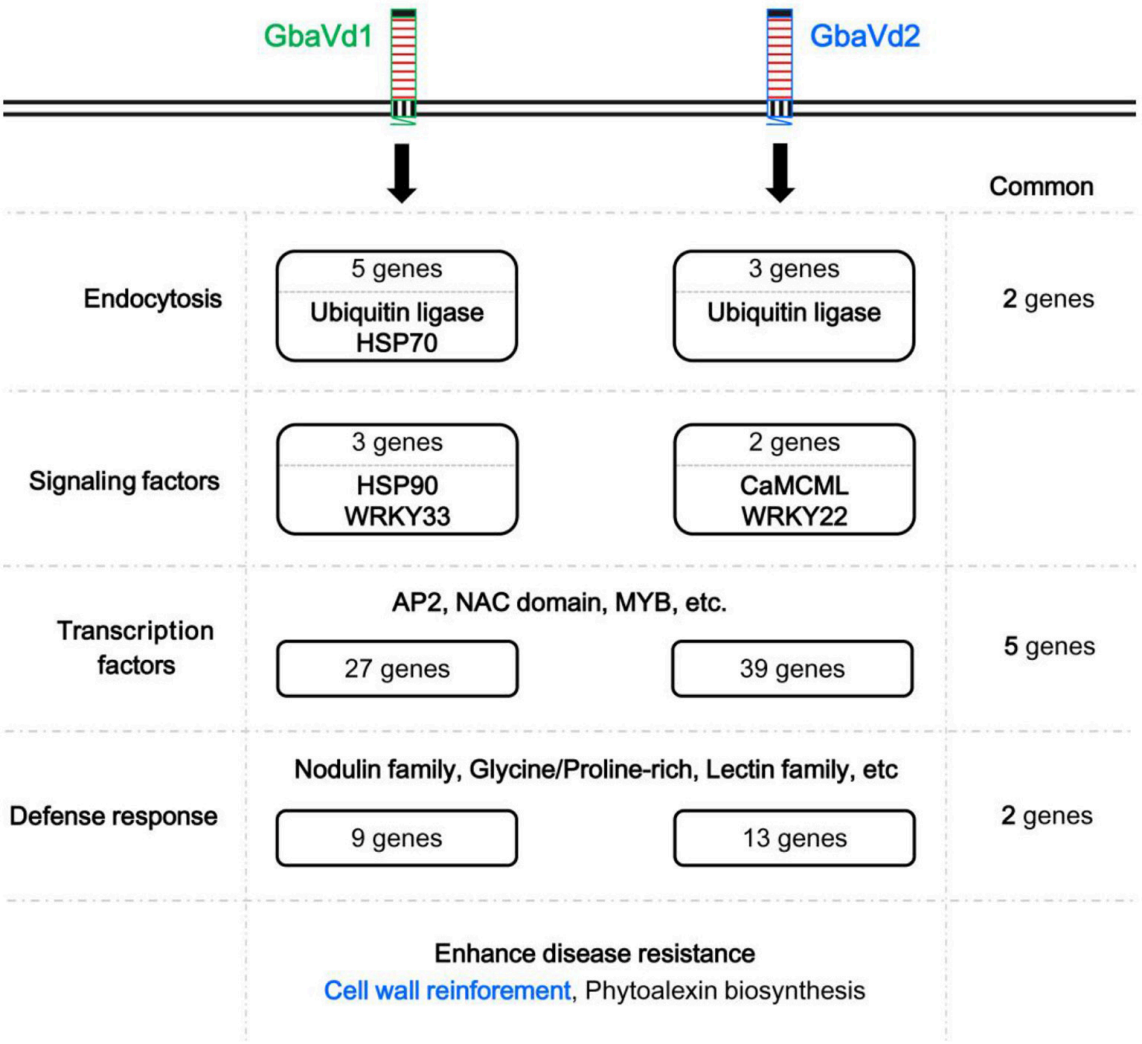
**Schematic overview of ***Verticillium*** wilt resistance mediated by ***GbaVd1*** and ***GbaVd2*****.

Although both *GbaVd1* and *GbaVd2* encode RLPs, the sequences and primary structures display divergence (Figures 1, 2), thus suggesting that the functions of *GbaVd1* and *GbaVd2* might diverge during plant-pathogen interactions. Microarray analysis revealed that at least four layers of defense responses were differentially activated after *GbaVd1* or *GbaVd2* overexpression in *Arabidopsis* (Figures 6, 7). Further analysis of the differentially expressed genes revealed that the defense responses mediated by *GbaVd1* and *GbaVd2* displayed significant differences that only five genes encoding transcription factors and two genes encoding cell wall-related proteins were common between the two transgenic lines (Figure 8). The activation of genes involved in endocytosis appeared to be similar between the *GbaVd1* and *GbaVd2* transgenic lines, with two common genes out of six differentially expressed genes, but more genes were activated in the *GbaVd1* transgenic plants (Figure 8). Together, these results suggested that *GbaVd1* and *GbaVd2* mediate different resistance signaling and activation of defense responses to confer *Verticillium* wilt resistance after interfamily overexpression in *Arabidopsis*.

## Discussion

*Verticillium* wilt resistance genes have been well studied in tomato plants, in which the *Ve* locus and the functional gene *Ve1* mediate the disease resistance to *V. dahliae* (Schaible et al., 1951; Fradin and Thomma, 2006; Fradin et al., 2009). Recently, several *Ve1* gene homologs, belonging to the RLP family, have also been isolated from cotton plants and demonstrated to be functional in *Verticillium* wilt resistance (Zhang et al., 2011, 2012; Yang et al., 2015; Chen et al., 2016). The *Verticillium* wilt resistance of cotton may be controlled by multi-dominant resistance genes. Previous studies have shown that the cotton genome encodes an extremely large RLP family protein that contains at least 144 RLPs in diploid cotton (Chen J. Y. et al., 2015), thus indicating that other RLP genes probably participate in *Verticillium* wilt resistance in cotton. In this study, two *RLP* genes, *GbaVd1* and *GbaVd2*, were cloned from the *Verticillium* wilt-resistant cultivar *G. barbadense* cv. Hai7124, which proved to be involved in Verticillium wilt resistance via the activation of diverse defense responses.

RLPs referred to as LRR-TMs, usually function as disease resistance genes in plants (Kruijt et al., 2005a). Like the primary structures of known RLPs (Dixon et al., 1996, 1998; Parniske et al., 1997; Vinatzer et al., 2001; Ron and Avni, 2004; Kruijt et al., 2005a; van der Hoorn et al., 2005), GbaVd1 and GbaVd2 contain signal peptides, LRR domains, transmembrane domains and small cytoplasmic tails (Figure 2; Figures S2, S3). GbaVd1 lack of any kinase characteristic in the small cytoplasmic domain. An orthologous gene of *GbaVd1, Gorai.003G112100.1* (Accession number: KJB19646.1), was designated as a disease resistance family protein_LRR due to the lack of kinase motif (Paterson et al., 2012), and we annotated it as RLP in cotton (Chen J. Y. et al., 2015). LRRs are typical domains of RLPs with the consensus sequence LxxLxxLxxLxLxxNxLxGxIPxx, and the number of LRRs may dictate the specific recognition of pathogen effectors (Jones et al., 1994; Jones and Jones, 1997; van der Hoorn et al., 2005). Moreover, the LRR domains of RLPs usually contain many potential *N*-glycosylation sites important to gene function; for example, *Cf-9* contains 22 PGSs, and most are glycosylated (van der Hoorn et al., 2005). GbaVd1 and GbaVd2 also contain different numbers of LRRs and potential *N*-glycosylation sites (Figure 2), thus possibly indicating that there are two kinds of RLPs in cotton. Like other RLPs, GbaVd1 and GbaVd2 contain a predicted small cytoplasmic tail that is necessary for signal transduction, as demonstrated by the interaction of the cytoplasmic peptide of *Cf-9* with *VAP27* and *CITRX* (Laurent et al., 2000; Nekrasov et al., 2006). Moreover, similar to the YXXø motif of LeEix2 and the KKX motif of Cf-4/9, which play important roles in the endocytotic process (Thomas et al., 1997; Ron and Avni, 2004; Sharfman et al., 2011), the cytoplasmic domain of GbaVd1 possesses a YXXø (YFRI) motif, and GbaVd2 contains a KKX motif (KKH) (Figure 2), which stimulates receptor-mediated endocytosis and mammalian cell-surface receptor degradation (Letourneur and Klausner, 1992; Benghezal et al., 2000; Bonifacino and Traub, 2003). Previous studies showed that the RLPs generally displayed significant divergence, which correspond to the fast evolution of pathogen (Kruijt et al., 2005a). Similarity, the sequence of allelic variants of GbaVd1 and GbaVd2 among the cotton species were also divergent, as the Cf-4 and Cf-9 in tomato (Kruijt et al., 2005b), but the polymorphisms do not obviously associate with the Verticillium wilt resistance (Figure S4). Thus, *GbaVd1* and *GbaVd2* are likely to encode typical RLPs with similar primary structures that contain extracytoplasmic LRR motifs and cytoplasmic tails.

RLPs play significant roles in plant defense against pathogens and plant development (Kruijt et al., 2005a). The interfamily transfer of tomato *Ve1*, a classical RLP, confers *Verticillium* wilt resistance in *Arabidopsis* (Fradin et al., 2011). Likewise, *Cf* proteins in tomato plants prevent the foliar leaf mold pathogen *Cladosporium fulvum* (Dixon et al., 1996, 1998; Parniske et al., 1997; Belfanti et al., 2004), and *HcrVf* proteins from *Malus domestica* enhance resistance to the scab fungus *Venturia inaequalis* (Vinatzer et al., 2001; Malnoy et al., 2008). Recently, several *Ve1* gene homologs identified in island cotton have been reported to confer resistance to *V. dahliae*, including *GbVe* (Zhang et al., 2011), *Gbve1* (Zhang et al., 2012), *Gbvdr5* (Yang et al., 2015), and *Gbvdr3* (Chen et al., 2016). In this study, *GbaVd1* and *GbaVd2* also conferred resistance to *V. dahliae* Vd991 stains (race 2) that lacks Ave1 (Yang et al., 2015), as demonstrated by VIGS in cotton and the interfamily transfer of the genes in *Arabidopsis* (Figures 3, 4). The ectopic overexpression of *GbaVd1* and *GbaVd2* was driven by the constitutive CaMV 35S promoter, which resulted in enhancing *Verticillium* wilt resistance in *Arabidopsis*. Previous studies showed that the ectopic expression of *Ve1* driven by the tomato native promoter is less effective than the CaMV 35S promoter in conferring *Verticillium* wilt resistance in *Arabidopsis* (Fradin et al., 2011). In addition, many other studies also found that overexpression of *RLP* genes from cotton by CaMV 35S promoter can significantly enhance the *Verticillium* wilt resistance in *Arabidopsis* (Zhang et al., 2011; Chen T. et al., 2015; Yang et al., 2015). Similarity, several resistance genes conferring resistance after overexpression in transgenic lines have been driven by constitutive promoter (Liu et al., 2005; Schoonbeek et al., 2015; Yeh et al., 2015). These results also support by previous findings that the *Ve*-like gene *Gbvdr5* conferred Verticillium wilt resistance in cotton and *Arabidopsis* transgenic plants (Yang et al., 2015), which only contains a residue divergence in GbaVd2 (Figure S3). In addition, several genes encoding RLP family proteins confer *Verticillium* wilt resistance, thus indicating that the inheritance of cotton resistance to *V. dahliae* may be controlled by multi-dominant resistance genes. Subsequently, except for the race 2 strain as the Vd991 used in this study, we need to further test the performance of *GbaVd1* or transgenic lines after inoculation with different *V. dahliae* genotypes, which facilitate us to understand the *Verticillium* wilt resistance functional *GbaVd1* and *GbaVd2*.

The genetics of RLP-mediated disease resistance is significantly associated with the endocytosis process, which plays a key role in plant-pathogen interactions (Gabriëls et al., 2006; Postma et al., 2016). Several RLPs contain an endocytosis motif (such as the YXXΦ endocytosis signature, where Φ represents an amino acid with a hydrophobic side chain, and X represents any amino acid) that can stimulate receptor endocytosis and initiate plant immunity (Jones et al., 1994; Thomas et al., 1997; Bonifacino and Traub, 2003; Ron and Avni, 2004; Sharfman et al., 2011). In addition, many signaling factors are recruited to participate in the resistance mediated by RLPs, which has been most extensively studied by exploiting the tomato *Cf* genes and the *Ve1* gene (Rowland et al., 2005; Gabriëls et al., 2006, 2007; González-Lamothe et al., 2006; Stulemeijer et al., 2007; van den Burg et al., 2008; Fradin et al., 2009). Similarly, GbaVd1 and GbaVd2 contain endocytosis motifs (Figure 2), and we found that the endocytosis process and signaling factors involved in the plant-pathogen interaction pathway were significantly activated after interfamily transfer into *Arabidopsis* (Figure 6), thereby resulting in an enhancement to *Verticillium* wilt resistance by initiating the defense response, as expected (Figure 7). Therefore, *GbaVd1* and *GbaVd2*, similarly to the known RLP genes, likely to provide resistance against *V. dahliae* by activation of the endocytosis process and recruitment of signaling factors.

Previous studies showed that EDS1, NRC1, ACIF, and MEK2 are required for the defense response mediated by Ve1 and Cfs. However, Serk3/Bak1 has not been tested for their requirement in Cfs signaling, while Sgt1 has not been tested for its requirement in Ve1 signaling, thus indicating that the RLP, Ve1 and Cf proteins, differentially require downstream signaling components (Fradin et al., 2009). Similarity, GbaVd1 and GbaVd2 mediated different regulation patterns and only a few genes were commonly regulated between the two transgenic plants (Figure 8). This means that the sequence and primary structures divergence of GbaVd1 and GbaVd2 may require different signaling factor (Figures 1, 2). It suggested that GbaVd2 (Gbvdr5) recognizes a new effector to activate the defense response due to the lack of Ave1 (race 2) in test strain Vd991 (de Jonge et al., 2012; Yang et al., 2015). We expected the defense mediated by GbaVd1 to display differences, due to the low sequence identity with GbaVd2 (Figures S3, S4). Plants generally employ several resistance genes to activate defense responses during pathogen infection (Wiesner-Hanks and Nelson, 2016), and several RLP genes have been identified in cotton that confer Verticillium wilt resistance (Zhang et al., 2011, 2012; Yang et al., 2015; Chen et al., 2016). Therefore, our study suggests that both *GbaVd1* and *GbaVd2* function as disease resistance proteins, but require different downstream signaling components to protect against *V. dahliae*.

RLP-mediated disease resistance activates a multilayered defense response, such as the production of reactive oxygen species (Piedras et al., 1998) and induction of the hypersensitive response (Ron and Avni, 2004), and drives phenylalanine-ammonia lyase gene expression (Gayoso et al., 2010). In this study, defense-related genes were also found to be activated in transgenic plants, including genes involved in enhancing the cell wall or phytoalexin biosynthesis (Figures 7A,B). Specifically, the genes encoding cell wall proteins were significantly affected in transgenic plants, thus resulting in reinforcement of plant cell walls in *Arabidops*is (Figure 7C). The cell wall is an important defensive structure for avoiding pathogen infection (Hückelhoven, 2007; Hématy et al., 2009). In the defense response mediated by *Cfs* in tomato plants, the plant cell wall-related genes are activated, thus leading to the reinforcement of the cell wall (Stergiopoulos and de Wit, 2009). In cotton, the resistant cultivar exhibited higher and earlier-induced levels of enzyme activity and lignin-like polymers compared with the susceptible cultivar (Smit and Dubery, 1997). Therefore, *GbaVd1* and *GbaVd2* probably possess the capacity to regulate the genes encoding cell wall proteins, resulting in enhancing *Verticillium* wilt resistance in *Arabidopsis*.

It is known that the *Ve*-like gene *Gbvdr5* confers resistance to *Verticillium dahliae* in transgenic *Arabidopsis* and upland cotton, which can be induced by hormones and activated defense response, including callose deposition, regulated defense-related genes expression, and hypersensitivity reaction (HR)-mimic cell death (Yang et al., 2015). Coincidently, the *Ve*-like genes cloned in our study, *GbaVd2* only displays a residue divergence at position 598 (Valine to leucine) compares to *Gbvdr5* (Figure S3). Our study confirmed that the polymorphism occurs between *GbaVd2* and *Gbvdr5*, and suggested that that *GbaVd2* and *Gbvdr5* are the same genes in *G. barbadense*, due to a random mutation between different starting materials. However, we found some novel defense response mediated by GbaVd2 (Gbvdr5) in this study, including differentially expressed the endocytosis process and signaling factor, and enhanced cell wall reinforcement (Figure 8), which facilitate us to further understand the mechanism of GbaVd2 (Gbvdr5) confers Verticillium wilt resistance in cotton.

In conclusion, two genes encoding RLPs, *GbaVd1* and *GbaVd2*, were cloned from the resistant cultivar of *G. barbadense* in this study. We revealed that *GbaVd1* and *GbaVd2* play essential roles in *Verticillium* wilt resistance, a result also demonstrated by VIGS in cotton and interfamily transfer into *Arabidopsis*. Similar to the *Verticillium* wilt resistance mediated by typical RLPs, genes associated with the endocytosis process, signaling factors, transcription factors and defense response were significantly activated after *GbaVd1* or *GbaVd2* overexpression, thereby resulting in cell wall reinforcement in *Arabidopsis*. In addition, microarray analysis revealed that *GbaVd1* and *GbaVd2* differentially mediated resistance signaling and activation of defense responses, corresponding to the divergence of *GbaVd1* and *GbaVd2* in terms of sequence, primary protein structure, and phylogeny.

## Author contributions

XD designed the experiments and revised the paper. JC performed the microarray analysis and gene cloning. NL, XM, TL, and DZ performed the experiment of gene function identification. JC, VG, and DZ wrote and critically reviewed this article.

### Conflict of interest statement

The authors declare that the research was conducted in the absence of any commercial or financial relationships that could be construed as a potential conflict of interest.

## Supplementary material

Sequence data from this article have been deposited with the GenBank data libraries under accession numbers GU299533 (*GbaVd1*) and GU299534 (*GbaVd2*). The microarray data are available via online.
