# Supplementary material for: The Ectopic Overexpression of the Cotton Ve1 and Ve2-Homolog Sequences Leads to Resistance Response to Verticillium Wilt in Arabidopsis
Source: Front Plant Sci. 2017 May 29;8:844. doi: 10.3389/fpls.2017.00844 (PMC5447073; doi:10.3389/fpls.2017.00844)
Supplement: Supplementary file 5 [file Presentation1.PDF]

Supplementary Material

The cotton genes *GbaVd1* and *GbaVd2* encode receptor-like proteins that confer resistance to *Verticillium* wilt

Running title:
Cotton receptor-like proteins

Authors:
Jieyin Chen[†,1], Nanyang Li[†,1], Xuefeng Ma[1], Vijai Kumar Gupta[2], Dandan Zhang[1], Tinggang Li[1], Xiaofeng Dai[1,1]*

[†] These authors contributed equally to this work

Institutional affiliation:

[1]Laboratory of cotton diseases， The Institute of Food Science and Technology, Chinese Academy of Agricultural Sciences, Beijing, China.

[2]Department of Chemistry and Biotechnology, ERA Chair of Green Chemistry, School of Science, Tallinn University of Technology, Tallinn, Estonia

*** Correspondence:** Xiao-Feng Dai: daixiaofeng_caas@126.com

1 Supplementary Figures

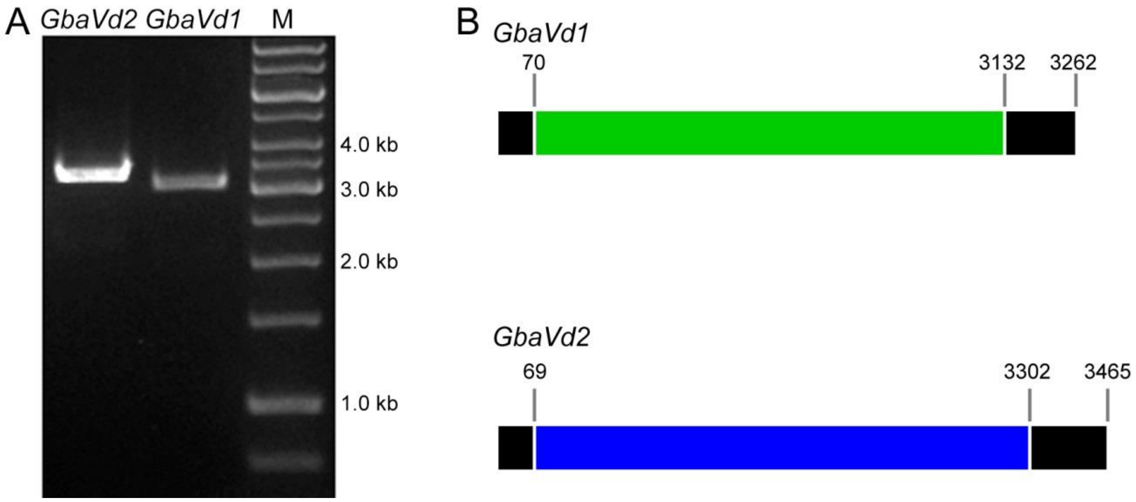

Figure S1. Cloning the full-length *GbaVd1* and *GbaVd2* genes. (**A**) PCR amplification of *GbaVd1* and *GbaVd2*. M, DNA marker. (**B**) Schematic of the ORFs in *GbaVd1* and *GbaVd2*. The ORFs of *GbaVd1* and *GbaVd2* are displayed in green and blue, respectively.

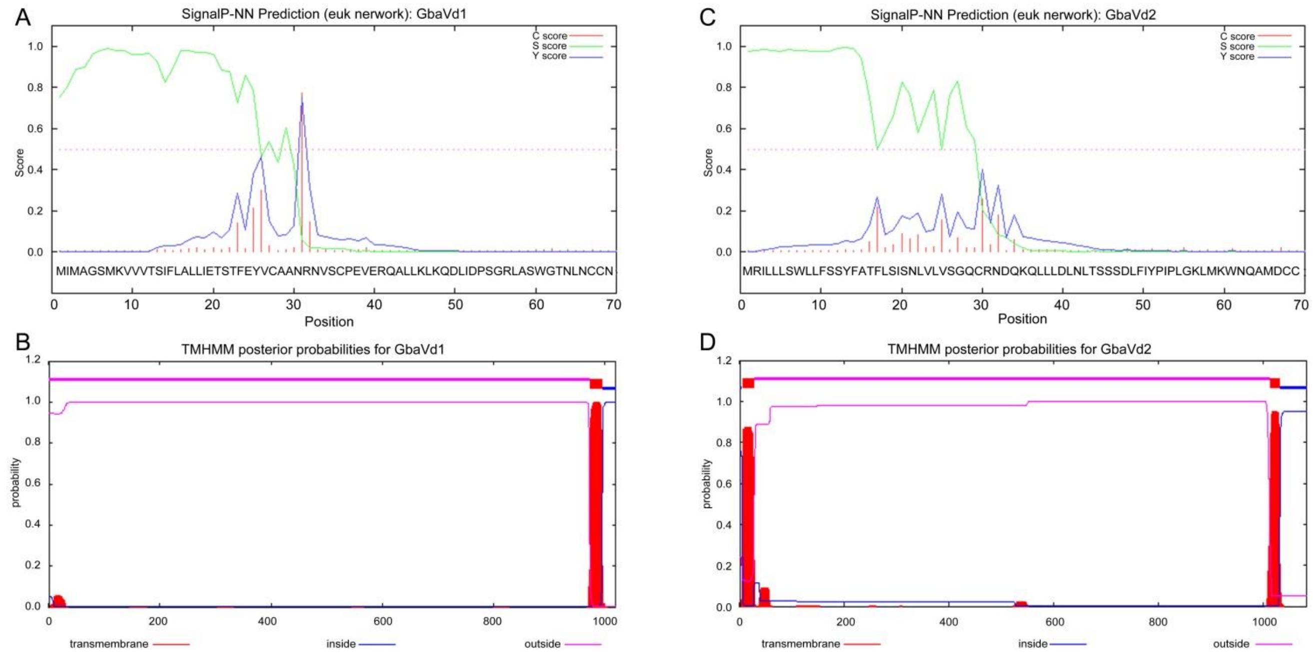

Figure S2. Bioinformatics analysis of the signal peptides and transmembrane domains of *GbaVd1* and *GbaVd2*. The signal peptides and transmembrane domains were predicted by using SignalP4.0 and THHMM 2.0 software, respectively.

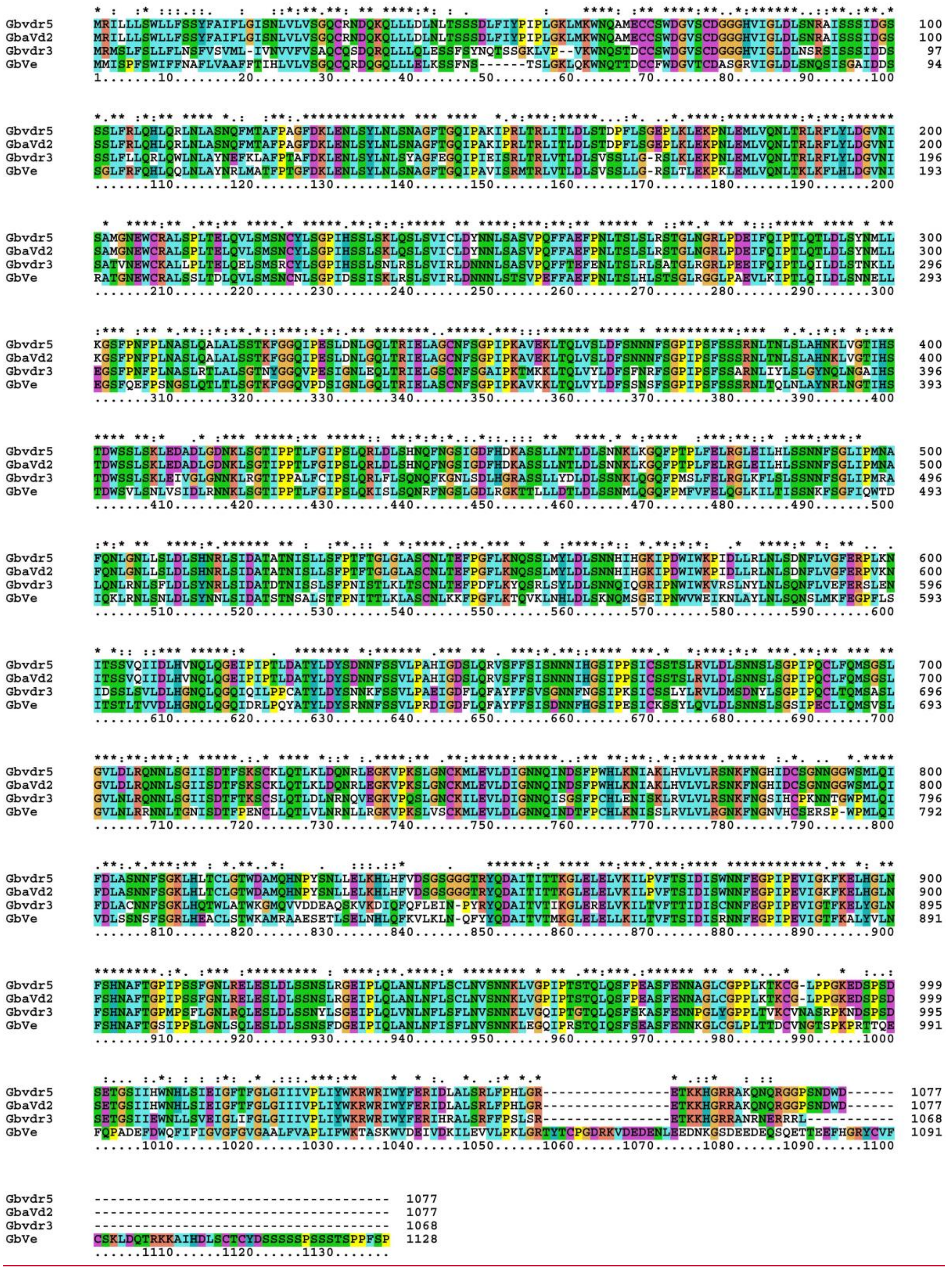

Figure S3. Alignment of GbaVd2 with Ve-like proteins from G. barbadense. ClustalX 1.83 software was used for the multiple sequence alignment. Gbvdr3 (Accession number: KP121692.1), Gbvdr5 (Accession number: KM066534.1), and GbVe (Accession number: EU855795.1).

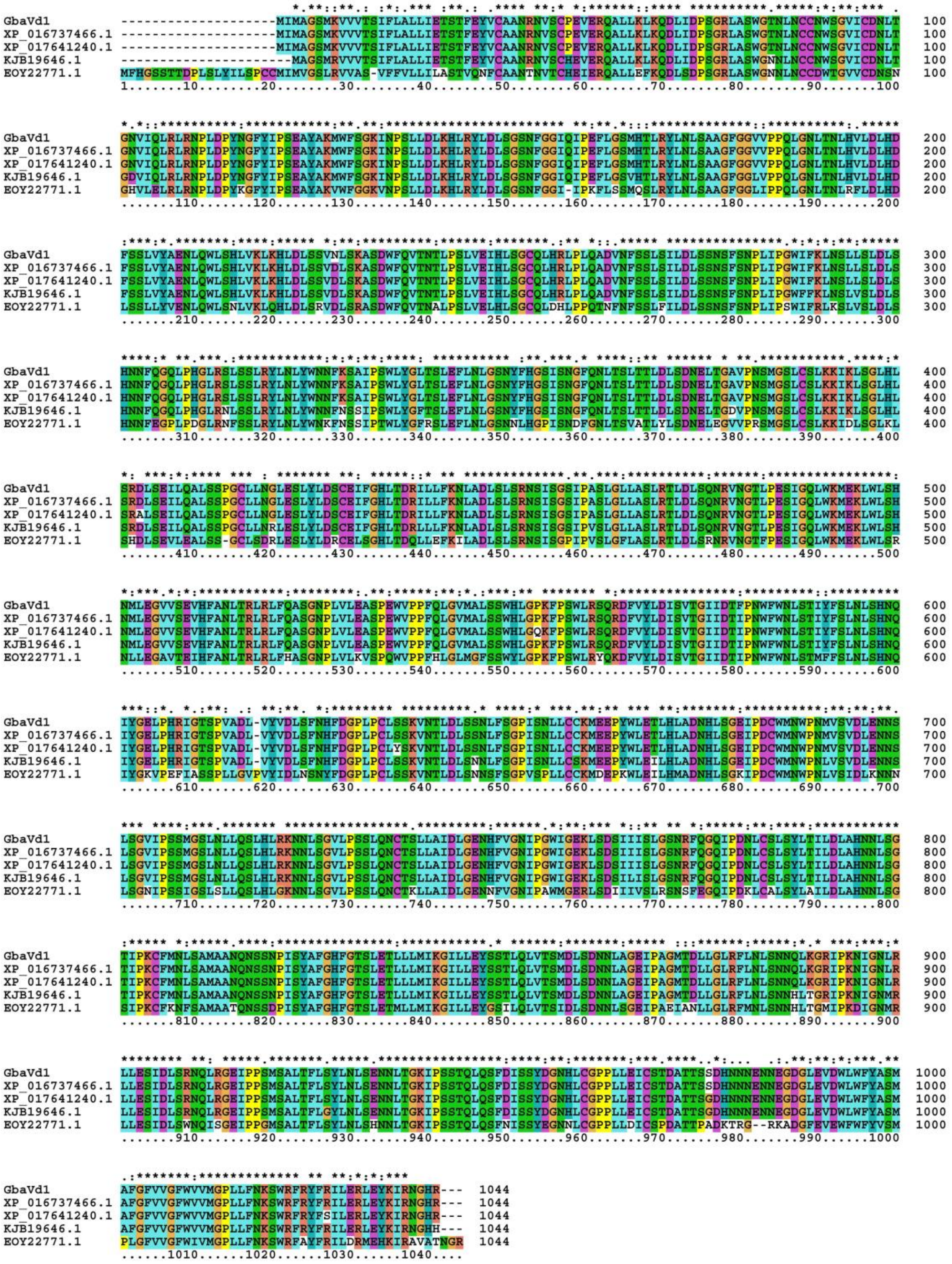

Figure S4. Alignment of *GbaVd1* with homolog RLP proteins. ClustalX 1.83 software was used for the multiple sequence alignment. *Gbvdr3* (Accession number: KP121692.1), *Gbvdr5* (Accession number: KM066534.1), and *GbVe* (Accession number: EU855795.1). XP_016737466.1, LRR receptor-like serine/threonine-protein kinase FLS2 from *Gossypium hirsutum*; XP_017641240.1, LRR receptor-like serine/threonine-protein kinase FLS2 from *Gossypium arboretum*; KJB19646.1,

hypothetical protein Gorai.003G112100.1 from *Gossypium raimondii*; EOY22771.1, disease resistance family protein/LRR family protein, *Theobroma cacao*.

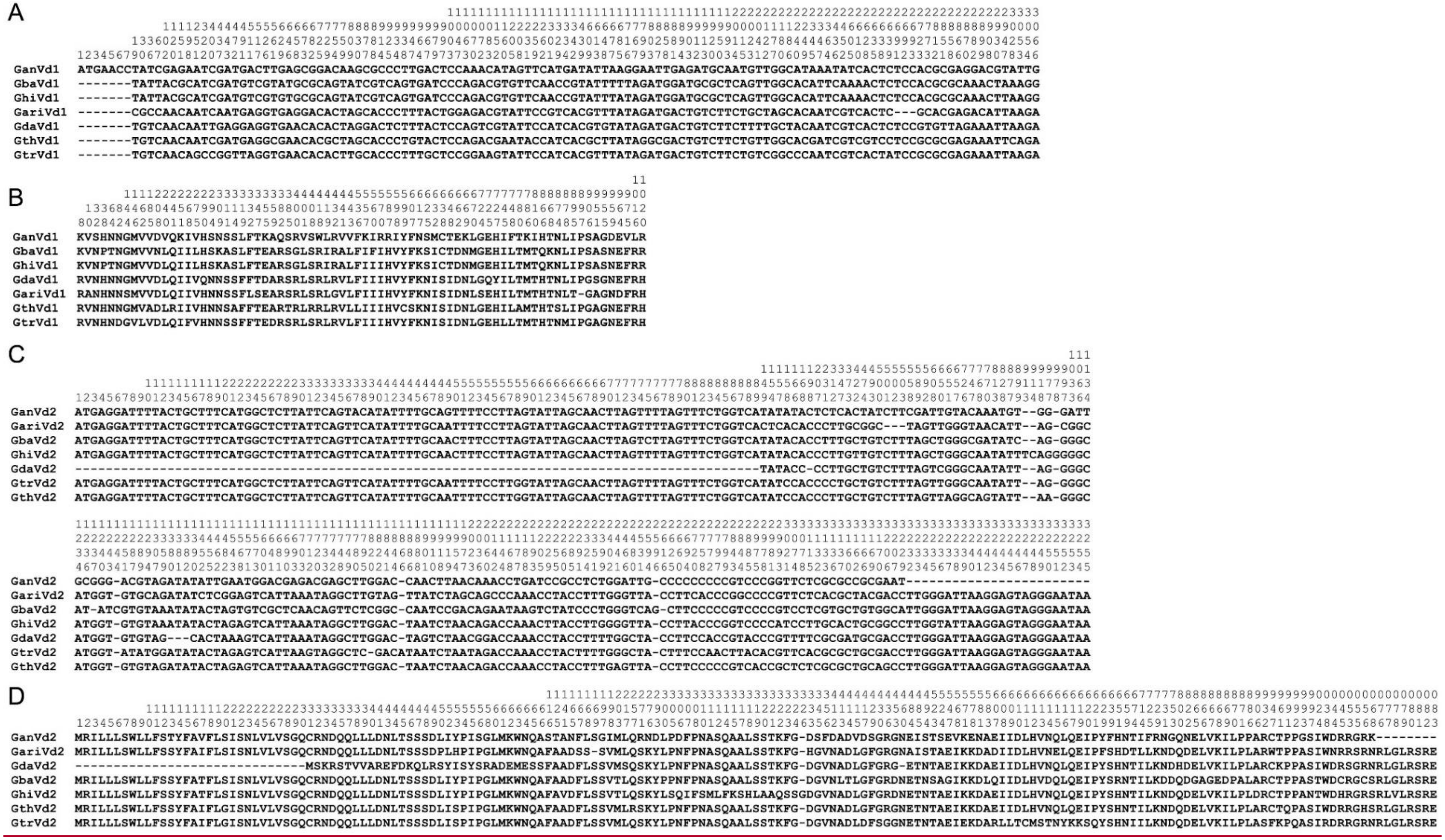

Figure S5. Alignment of the *GbaVd1* and *GbaVd2* allelic genes from six cotton species. ClustalX 1.83 software was used for the multiple sequence alignment, all sequences are compared with the reference GbaVd1 or GbaVd2. (**A**), DNA or (**B**), protein sequence of GbaVd1, and (**C**), DNA or (**D**), protein sequence of GbaVd2. Only residues that deviate from these reference sequences are shown in the alignment, the deletion are indicated by a dash (-).*GanVd1*/*GanVd2*: *Gossypium anomalum*; *GtrVd1*/*GtrVd2*: *Gossypium trilobum*; *GariVd1*/*GariVd2*: *Gossypium aridum*; *GdaVd1*/*GdaVd2*: *Gossypium davidsonii*; *GthVd1*/*GthVd2*: *Gossypium thurberi*; *GhiVd1*/*GhiVd2*: *Gossypium hirsutum*.

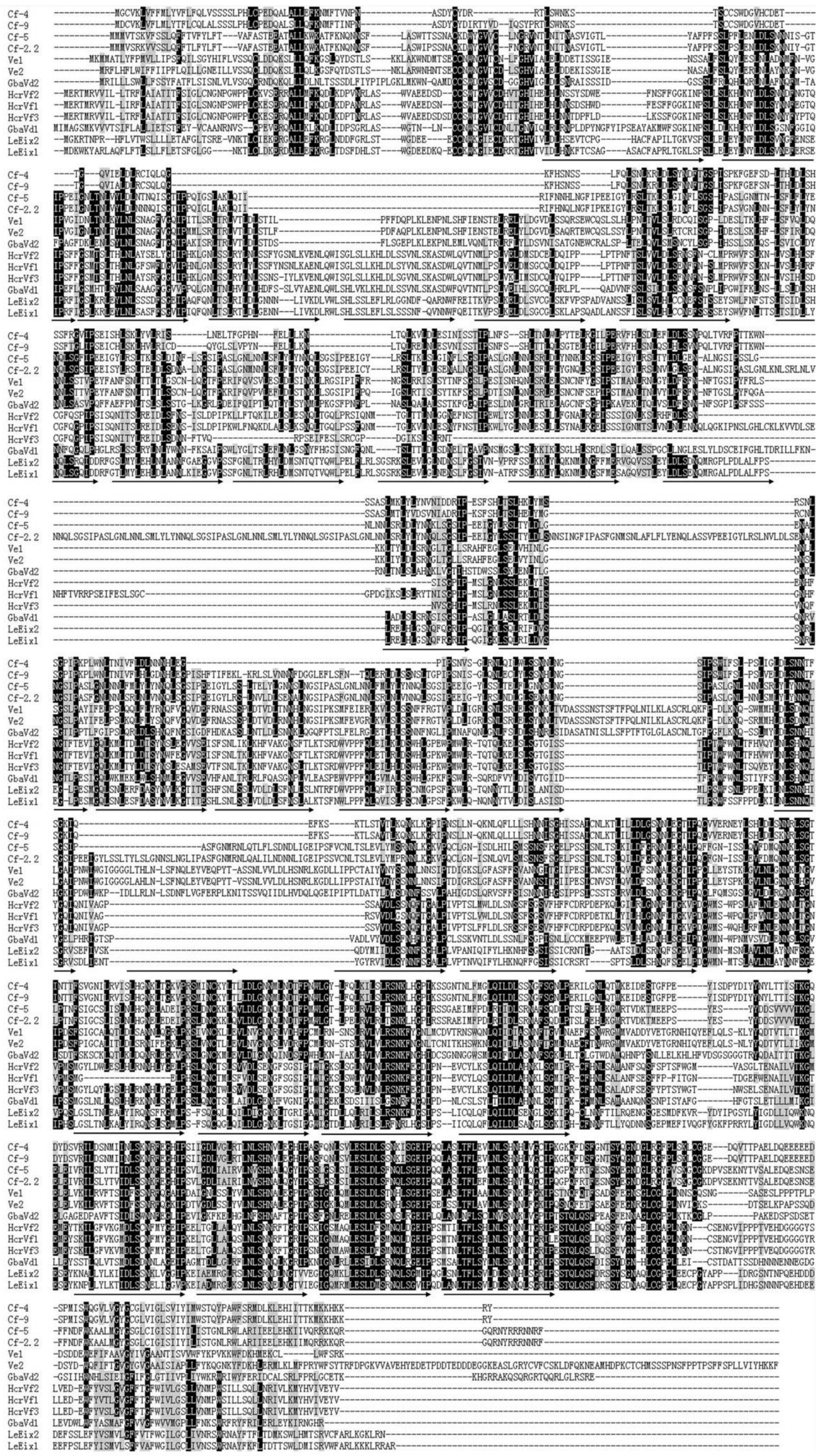

Figure ~~S3~~S6. Alignment of *GbaVd1* and *GbaVd2* with known RLPs. ClustalX 1.83 software was used for the multiple sequence alignment, which was exported by using Boxshade 3.21. The LRR structures are highlighted with arrows. *Cf-2.2*, U42445; *Cf-5*, AF053993; *Cf-4*, AJ002235; *Cf-9*, AJ002236; *HcrVf1*, AJ297739; *HcrVf2*, AJ297740; *HcrVf3*, AJ297741; *LeEix1*, AY359965; *LeEix2*, AY359966; *Ve1*, AF365929; *Ve2*, AF272366; *GbaVd1*, GU299533; *GbaVd2*, *GU299534*.

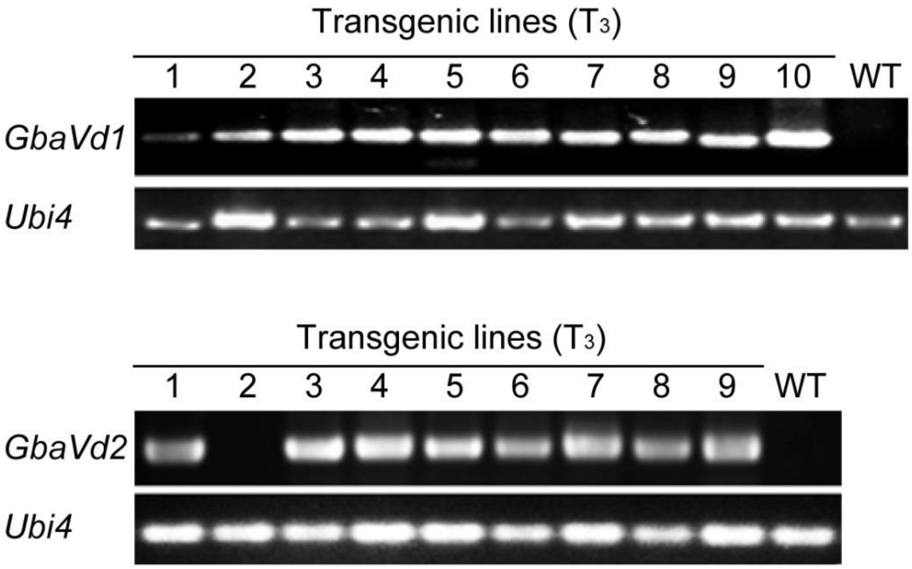

Figure ~~S4~~S7. Identification of successful *GbaVd1* and *GbaVd2* expression in *Arabidopsis* transgenic lines by RT-PCR. Ubiquitin 4 (*Ubi4*) was used as a control. WT, wild-type *Col-0*.

2 Supplementary Tables and Data

Supplementary Table S1 to S4 and Supplementary Data S1 for this article can be found online.
